# miR-96-5p antagonizes FOXQ1-driven WNT/β-catenin signaling to inhibit triple-negative breast cancer

**DOI:** 10.1038/s41598-025-34859-7

**Published:** 2026-01-04

**Authors:** Zhuoran Zhang, Chenshan Zhang, Yuxuan Meng, Xianyu Zhu, Jianying Yang, Bing Ran, Zhonglin Gan, Kejiang Wang, Mao Yang, Pinjun Lu, Sakorn Pornprasert, Tao He, Yan Lin

**Affiliations:** 1 Institute for Cancer Medicine, School of Basic Medical Sciences, Southwest Medical University, No.1 Section 1, Xiang Lin Road, Longmatan District, Luzhou, 646000 Sichuan China; 2 Academic Affairs Office and Higher Education Research Institution, Southwest Medical University, Luzhou, 646000 Sichuan China; 3 Department of Physiology, School of Basic Medical Sciences, Southwest Medical University, Luzhou, 646000 Sichuan China; 4 Functional Laboratory, School of Basic Medical Sciences, Southwest Medical University, Luzhou, 646000 Sichuan China; 5 Department of Laboratory Medicine, Meishan City People’s Hospital, Meishan, 620000 Sichuan China; 6 Department of Medical Technology, Faculty of Associated Medical Sciences, Chiang Mai University, Chiang Mai, 50200 Thailand

**Keywords:** Triple-negative breast cancer, FOXQ1, miR-96-5p, WNT2, Wnt/β-catenin signaling, Feedback loop, Biogeochemistry, Breast cancer, Cancer, Targeted therapies

## Abstract

FOXQ1 overexpression and Wnt/β-catenin pathway hyperactivation are implicated in TNBC, but their functional interconnection is poorly defined. The expression pattern, clinical significance, and oncogenic role of FOXQ1 were systematically assessed using data from The Cancer Genome Atlas (TCGA) combined with RT-PCR, western blotting, and Transwell assays. To explore the transcriptional regulation of FOXQ1 on WNT2, we integrated bioinformatic predictions with analyses of the CCLE dataset, RT-qPCR, western blotting, and luciferase reporter assays. The functional interplay within the FOXQ1-WNT2-β-catenin signaling axis was evaluated through immunofluorescence staining, TOP/FOP flash reporter assays, and pharmacological inhibition using the Wnt pathway inhibitor IWP-2. miR-96-5p was identified through multi-database screening and experimentally validated by 3’UTR reporter assays, functional experiments, and in vivo xenograft models. FOXQ1 is upregulated in TNBC and correlates with poor patient survival (HR = 1.34, *p* = 0.041). It promotes epithelial-mesenchymal transition (EMT), cell migration, and invasion in TNBC. Mechanistically, FOXQ1 directly transactivates WNT2, leading to enhanced nuclear translocation and transcriptional activation of β-catenin. Although IWP-2 inhibits β-catenin activation, it does not prevent FOXQ1-induced upregulation of WNT2, indicating that FOXQ1 acts both upstream of and independently within the Wnt signaling pathway. Furthermore, miR-96-5p directly targets the 3’UTR of FOXQ1, is associated with an improved prognosis (HR = 0.82, *p* = 0.048), and suppresses TNBC tumor growth and aggressiveness in vitro and in vivo. In conclusion, we have identified a FOXQ1-WNT2-β-catenin positive feedback loop as a critical driver of TNBC pathogenesis and propose miR-96-5p as a promising therapeutic strategy to suppress this signaling pathway.

**Supplementary Information:**

The online version contains supplementary material available at 10.1038/s41598-025-34859-7.

## Introduction

Breast cancer is the leading cause of cancer incidence and cancer-related mortality among women, with 2.3 million new cases and 665,000 deaths reported globally in 2022^1^. Triple-negative breast cancer (TNBC), accounting for 15%-20% of all breast cancers^2^, is distinguished by its highly aggressive nature. Due to the absence of estrogen receptor (ER), progesterone receptor (PR), and human epidermal growth factor receptor 2 (HER2) expression, TNBC does not respond to endocrine or HER2-targeted therapies. Moreover, the scarcity of effective molecular targets further restricts treatment options^3^, leading to a median overall survival of only 12 to 18 months for patients with metastatic TNBC^4^. Consequently, TNBC is considered one of the subtypes with the poorest prognosis. Identifying viable molecule targets continues to be a critical challenge in the clinical management of TNBC.

FOXQ1, also known as HNF-3/HFH homolog-1 (HFH-1), is a member of the forkhead protein family. Initially identified as a transcription factor, it plays a vital role in the development of hair follicles^5,6^. Later, FOXQ1 was recognized as an oncogene because numerous studies have demonstrated that FOXQ1 regulates various biological processes, including tumor proliferation, inflammation, epithelial-mesenchymal transition (EMT), migration, invasion, and angiogenesis^7–9^. In breast cancer, FOXQ1 was required for FGFR1 activation and upregulated proliferation^10^. It recruits the MLL complex to activate the transcription of genes associated with EMT and promote breast cancer metastasis^8^. Moreover, FOXQ1 encourages aggressiveness and radioresistance in breast cancer by interacting with a nuclear isoform of RAPH1^11^. Consequently, FOXQ1 induction has been recognized as an independent prognostic factor for gastric and colorectal cancer and a promising therapeutic target for metastatic cancer treatment^12^.

The Wnt/β-catenin signaling pathway, initially identified by Nusse and Varmus in 1982^13^, is a highly conserved mechanism that plays a critical role in regulating stem cell differentiation^14^, organ development^15^, and tissue regeneration^16^. Hyperactivation of the Wnt/β-catenin signaling pathway is intricately linked to breast cancer initiation, progression, and malignant transformation^17^. Therefore, the Wnt/β-catenin pathway is acknowledged as a crucial signaling pathway for tumor progression. Our prior research revealed a positive correlation between FOXQ1 expression and the activation of the FGFR1/Wnt/β-catenin signaling pathway in breast cancer^18^. Moreover, Peng et al.^19^ illustrated that silencing FOXQ1 in colorectal cancer inhibits the nuclear translocation of β-catenin, consequently attenuating Wnt signaling activity. These findings collectively suggest that FOXQ1 is a critical regulator of the Wnt/β-catenin signaling pathways, which are intricately involved in breast cancer progression. Nevertheless, the exact mechanisms through which FOXQ1 modulates this pathway in TNBC remain largely elusive. Additionally, the potential negative regulators that interact with FOXQ1 and the Wnt/β-catenin signaling pathways warrant further exploration.

MicroRNAs (miR) are a group of non-coding single-stranded RNA molecules composed of 19–25 nucleotides^20^. It can recognize and directly bind to the complementary site within the 3’-untranslated region (UTR) of target genes’ mRNA, resulting in degradation or transcriptional repression. Consequently, they exhibit potent regulatory capabilities over gene expression. Through their targeted interaction with specific molecules, several miRNAs have been identified as potential suppressors of breast cancer. For instance, miR-142-3p was up-regulated in breast cancer tissue and suppressed breast cancer malignancy by targeting HMGA2^21^. Xu et al.^22^ reported that miR-193, as an oncogene, enhanced cell EMT and proliferation through the ING5/PI3K/AKT signal pathway in TNBC. These findings highlight the potential of miRNAs for therapeutic interventions in TNBC.

This study systematically investigated the functional interplay among FOXQ1, WNT2, and the Wnt/β-catenin signaling pathway in TNBC cells by integrating population-based data, bioinformatic analyses, and a comprehensive series of in vitro and in vivo experiments. Furthermore, we identified multiple microRNAs (miRNAs) targeting FOXQ1 through bioinformatic prediction and functionally validated the role of miR-96-5p in suppressing FOXQ1-driven tumorigenesis in TNBC. Our findings uncover a novel molecular mechanism underlying TNBC progression and suggest potential therapeutic strategies for its treatment.

##  Materials and methods

### Cell culture

Human TNBC cell lines (MDA-MB-231 and Hs578T) were purchased from the Shanghai Cell Bank(Shanghai, China) MDA-MB-453, BT474, MCF-7, T47D, and HEK293T cells were provided by the Institute for Cancer Medicine, School of Basic Medical Sciences, Southwest Medical University. The cells were cultured in Dulbecco’s Modified Eagle’s Medium (DMEM) (Cytiva HyClone, USA) supplemented with 10% fetal bovine serum (FBS, PAN, Germany) at 37 °C in a tissue culture incubator with 5% CO_2_.

### Construction of cell lines that stably express FOXQ1

The FOXQ1-expressing plasmid pEZ-LV201 (EX-Y5225-LV201) and its empty control vector LV201CT (EX-NEG-LV201) were purchased from GeneCopoeia (Rockville, USA). The cells were transfected with each plasmid DNA using Lipofectamine 8000 (c0533, Beyotime Biotechnology, Shanghai, China) at a DNA-to-transfection reagent ratio of 1:1.25. Five hours after transfection, the medium was replaced with fresh medium containing 10% FBS. Once significant green fluorescence was observed in the cells, the culture medium was changed to a growth selection medium containing 2.25 µg/mL puromycin. After 2 weeks of selection, the enhanced green fluorescent protein (eGFP)-positive cells were isolated from the surviving population by flow cytometry and expanded. Then, the FOXQ1 messenger RNA (mRNA) and protein levels were analyzed using real-time quantitative reverse transcription polymerase chain reaction (RT-qPCR) and western blotting, respectively.

### Oligonucleotide transfection

Two small interfering RNAs (siRNAs; si-1# and si-2#) targeting FOXQ1 and a negative control were obtained from Sangon Bioengineering Co., Ltd. (Shanghai, China). The miR-96-5p mimic and its negative control vector were sourced from General Bio Co (Anhui, China). These oligonucleotides were transfected into cells using Lipofectamine 8000 at an oligonucleotide to transfection reagent ratio of 1:1.25. The knockdown efficiency of the FOXQ1 protein was evaluated with western blotting, and the levels of miR-96-5p in cells were assessed with RT-qPCR.

### Western blotting

Protein was extracted with radioimmunoprecipitation assay (RIPA) lysis buffer (Beyotime Biotechnology) containing 1% ethylenediaminetetraacetic acid (EDTA)-free protease inhibitor cocktail (MedChemExpress, USA), and quantified with a BCA protein assay kit (Beyotime Biotechnology). Forty micrograms of protein were separated by 10% or 12% sodium dodecyl sulfate-polyacrylamide gel electrophoresis and transferred to nitrocellulose membranes (P-N66485, PALL, USA). After incubating with 5% bovine serum albumin (BIO FROXX, USA) dissolved in Tris-buffered saline (pH 8.3) and 0.1% Tween-20 (TBS-T) at room temperature for 1 h to block non-specific protein binding, the membranes were incubated with primary antibodies specific to FOXQ1 (1:250,sc-166265, Santa-Cruz, USA), WNT2 (1:1000, 27214-1-AP, Proteintech, USA), phosphorylated GSK-3β (1:1000, AF5830, Beyotime Biotechnology), GSK-3β (1:1000, AG751, Beyotime Biotechnology), β-catenin (1:5000,51067-2-AP, Proteintech), and GAPDH (1:5000, 60004-1-Ig, Proteintech) overnight at 4 °C. Subsequently, the membranes were incubated for 2 h at room temperature with a fluorescence-labelled secondary antibody: DyLight 800-conjugated anti-rabbit IgG (5151P, Cell Signaling, USA) or DyLight 800-conjugated anti-mouse IgG (5257P, Cell Signaling, USA). After this incubation, the membranes were washed three times with TBS-T. The Odyssey Imaging System (LI-COR, NE, USA) was utilized to monitor the fluorescence intensity of the bands. Band intensity was analyzed using the ImageJ software (National Institutes of Health, Bethesda, MD, USA). GAPDH served as a loading control for total proteins.

### RT-qPCR

Total RNA was extracted from cells using Trizol (vazyme, China), and HiScript III All-in-one RT SuperMix Perfect for qPCR(R333-01, Vazyme, China) and miRNA 1st Strand cDNA Synthesis kit(MR201-, Vazyme, China) were used to reversely transcribe RNA into cDNA for mRNA and miRNA, respectively. Real-time PCR was performed with cDNA, primers, and the Taq Pro Universal SYBR qPCR Master Mix (Vazyme). β-actin or U6 served as the internal reference. The relative levels of FOXQ1 and miRNAs were calculated according to the 2^−ΔΔCt^ method. The following primers were used: FOXQ1: forward, 5′-GCGGACTTTGCACTTTGAA-3′, and reverse, 5′-TTTAAGGCACGTTTGATGGA-3′; Vimentin: forward,5′-GAGAACTTTGCCGTTGAAGC-3′, and reverse, 5′-GCTTCCTGTAGGTGGCAATC-3′; miR-96-5p: forward, 5′-GCTTTGGCACTAGCACATTTTTGCT-3′, and reverse, the Novozyme qPCR Universal Primer Q(Vazyme); U6: forward 5′-CTCGCTTCGGCAGCACA-3′, and reverse, 5′-AACGCTTCACAATTTGCGT-3′; and β-actin: forward, 5′-CCTAGAAGCATTTGCGGTGG-3′, and reverse 5′-GAGCTACGAGCTGCCTGACG-3′.

### Transwell migration and invasion assay

A transwell assay was used to measure the migration and invasion ability of TNBC cells. For this assay, a 24-well chamber (Corelle, Kenny Bunker) was pre-coated with Matrigel (BD, Franklin Lakes, NJ, USA) diluted in serum-free medium at a ratio of 1:8 (for the invasion assay) or without Matrigel (for the migration assay). Approximately 30,000 cells were mixed with 300 µL of serum-free DMEM and placed in the upper chamber; 500 µL of DMEM containing 20% serum was added to the lower chamber. After culturing for 24 h, the migratory or invasive cells were washed with phosphate-buffered saline (PBS), fixed with methanol, stained with 0.1% crystal violet solution, and counted under a microscope.

### Luciferase reporter assay

Pmir-GLO (Hebio, Shanghai, China) is a plasmid containing both firefly luciferase and *Renilla* luciferase. The 3′ untranslated region (UTR) of FOXQ1 mRNA was amplified with PCR and inserted into the downstream area of the SV40 promoter-driven *Renilla* luciferase cassette in the Pmir-GLO plasmid. The FOXQ1 binding sequence region was antisense-mutated, and the mutant Pmir-GLO plasmid (MUT) was constructed in the same way. Next, HEK293T cells were transfected with miR-96-5p mimics and NC, as well as Pmir-GLO. After 48 h, the cells were lysed and collected for measurement of luciferase activity by the dual-luciferase assay system (Beyotime Biotechnology), following the manufacturer’s protocols.

### TOP/FOP-Flash reporter assay

To evaluate the transcriptional activation of the β-catenin-mediated Wnt signaling pathway, HEK293T cells were co-transfected with either the TOP-Flash or FOP-Flash firefly luciferase reporter plasmid, along with the SV40-Renilla luciferase plasmid (Beyotime Biotechnology). The TOP-Flash reporter system, which contains functional TCF/LEF binding sites, was used to assess β-catenin transcriptional activity. As a negative control, the FOP-Flash construct, containing mutated TCF/LEF binding sites, was used in parallel experiments. Following transfection, cells were treated for 24 h with one of the following: vehicle control, the Wnt inhibitor IWP-2^23^ (8 µM), FOXQ1 overexpression, or FOXQ1 combined with IWP-2. Luciferase activity was measured using the Dual Luciferase Assay Reporter System (Beyotime Biotechnology). In all assays, Renilla luciferase activity was measured concurrently and used for normalization to account for variations in transfection efficiency and cell viability. The concentration of IWP-2 (8 µM) was selected based on a CCK-8 cytotoxicity assay, which confirmed minimal cytotoxicity under the experimental conditions. Additionally, the efficacy of IWP-2 at this concentration in suppressing Wnt/β-catenin signaling was independently validated using the TOP/FOP-Flash reporter assay (see Supplementary Figure S1).

### Animal experiments

All animal experimental procedures adhered to the Animal Research: Reporting of In Vivo Experiments (ARRIVE) guidelines. The experimental protocol was reviewed and approved by the Experimental Animal Ethics Committee of Southwest Medical University under protocol No. 20220624-010. Female BALB/c-nu mice (3–4 weeks old) were purchased from Chengdu Pharmachem Biotechnology Co., Ltd. (Chengdu, China). On day 1, before surgery, mice were anesthetized via intraperitoneal injection with 3% sodium pentobarbital at a dose of 1.5 mg/kg body weight. Ten million Hs578T cells that stably overexpress FOXQ1 or control cells were injected into the mammary fat pad of each mouse. Each group consisted of 10 mice. After 9 days, when most tumors had reached approximately 50 mm^3^ in volume, the tumor formation rate was recorded. Tumor volume was calculated using a caliper with the modified ellipsoidal formula: volume (mm^3^) = (width×width×length)/2. Mice bearing FOXQ1-overexpressing tumors were then randomly divided into two groups and received either miR-96-5p agonist or saline injection intratumorally every 2 days. Control mice that developed tumors (*n* = 5) received intratumoral saline injection every 2 days, while those that did not develop tumors were given saline injections in the mammary fat pad. On day 20, all mice were humanely euthanized via excessive CO2 inhalation according to the approved protocol No. 20220624-010. Tumor size and weight were subsequently measured. All procedures, including anesthesia, tumor inoculation, and euthanasia, were designed to minimize animal suffering.

### Immunohistochemistry

Fresh tumor samples were fixed, embedded with paraffin, and sectioned, and the endogenous peroxidase activity was inactivated, as previously described^10^. The sections were incubated with goat-serum-containing blocking solution (Cat #abs933, Absinand) and then with the following antibodies overnight at 4 °C: rabbit anti-FOXQ1 antibody (1:250, sc-166265, Santa-Cruz), rabbit anti-KI67 antibody (1:1000, GB111499, Servicebio, China), and rabbit anti-vimentin (1:800, GB11192, Servicebio). After three washes with PBS, the cells were incubated with horseradish peroxidase-labelled secondary antibodies of the corresponding species for 50 min. After incubation with 3,3’-diaminobenzidine (DAB) to develop a colored product, the sections were stained with hematoxylin for 3 min, dehydrated, sealed, and examined with a microscope. The FOXQ1 and vimentin immunoreactivity scores, as well as the percentage of Ki67-positive cells, were calculated as described previously^10^.

### Software and databases

Population data were sourced from The Cancer Genome Atlas (TCGA) (https://www.cancer.gov/ccg/research/genome-sequencing/tcga). The Kaplan-Meier Plotter (https://kmplot.com/analysis/index.php?p=service) was applied to assess the association between FOXQ1 expression levels and both overall survival and recurrence-free survival. Potential downstream targets of FOXQ1 were predicted using TRANSFAC (https://genexplain.com/transfac/) and TRRUST (https://www.grnpedia.org/trrust/). Gene Ontology (GO) enrichment analysis was performed using R software (version 4.3.1) with the clusterProfiler package (v4.10.0) to identify biologically relevant pathways. Data visualization was conducted using the ggplot2 package (v3.4.4). The Starbase database (https://starbase.sysu.edu.cn/) was used to evaluate the correlation between FOXQ1 and WNT2 mRNA expression. At the same time, the Cancer Cell Line Encyclopedia (CCLE) (https://sites.broadinstitute.org/ccle) was utilized to investigate the relationship between FOXQ1 expression and WNT2 copy number variations. Putative microRNAs targeting FOXQ1 were identified through TargetScan (https://www.targetscan.org/vert_80/) and miRDB (https://mirdb.org/).

### Statistical analysis

Each experiment was repeated more than three times, and the graphs in the figures show the representative results of these experiments. Statistical analysis was performed using GraphPad Prism 9 (GraphPad Software, San Diego, CA, USA). The data are presented as the mean ± standard deviation. The difference between the two groups was assessed using an unpaired Student’s t-test, while the difference among multiple groups was evaluated using a one-way analysis of variance (ANOVA).

##  Results

### FOXQ1 is significantly upregulated in TNBC and is associated with an unfavorable prognosis

**Fig. 1:**
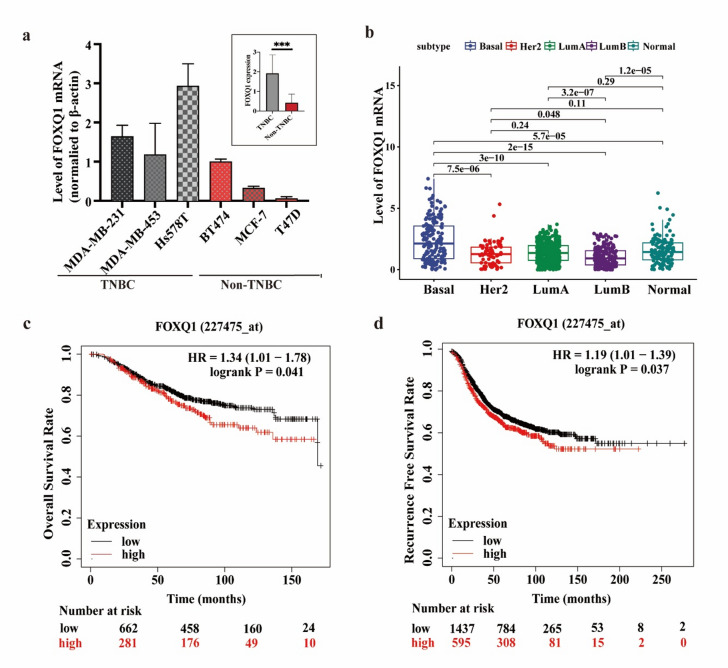
FOXQ1 is significantly upregulated in TNBC and is associated with an unfavorable prognosis. (**a**) The levels of FOXQ1 mRNA were assessed in several triple-negative and non-triple-negative breast cancer cell lines derived from our laboratory. FOXQ1 mRNA expression was quantified using real-time quantitative PCR (qPCR) and normalized to β-actin expression. (**b**) The expression levels of FOXQ1 mRNA were analyzed in tumor tissues from 1079 patients with various breast cancer subtypes obtained from the TCGA database. Intergroup differences were assessed using Tukey’s multiple comparisons test following one-way ANOVA. *P* < 0.05 presents a statistically significant difference. (**c**) Kaplan-Meier-plotter analysis of the overall survival rate of breast cancer patients with different FOXQ1 expression levels (*n* = 943). All data from https://kmplot.com/analysis/. (**d**) Kaplan-Meier plotter analysis of the recurrence-free survival rate of breast cancer patients with different FOXQ1 expression levels (*n* = 2032).All data from https://kmplot.com/analysis/.

To investigate the expression pattern of FOXQ1, we conducted RT-qPCR to examine the FOXQ1 mRNA levels across various breast cancer cell lines. There was a significantly higher average level in three TNBC cell lines, specifically MDA-MB-231, MDA-MB-453, and Hs578T, compared with non-TNBC cell lines, such as BT474, MCF-7, and T47D, all of which were cultured in our laboratory (Fig. 1a). Additionally, we analyzed the FOXQ1 mRNA levels in 1079 breast cancer samples from the TCGA database. The FOXQ1 mRNA levels were notably higher in the basal-like subtype compared with the HER2, luminal A, and luminal B subtypes, as well as normal tissue samples (*P* < 0.05) (Fig. 1b). Furthermore, we investigated the correlation between FOXQ1 expression and patient outcomes using data from the TCGA database. Kaplan-Meier Plotter analysis demonstrated a significant correlation between increased FOXQ1 expression and both unfavorable overall survival (HR = 1.34, *P* = 0.041) and poorer recurrence-free survival (HR = 1.19, *P* = 0.037) (Fig. 1c and d). These findings suggest that FOXQ1 expression is significantly upregulated in TNBC and is associated with a poorer prognosis.

### FOXQ1 plays a crucial role in enhancing the migration and invasion capabilities of TNBC cells

**Fig. 2:**
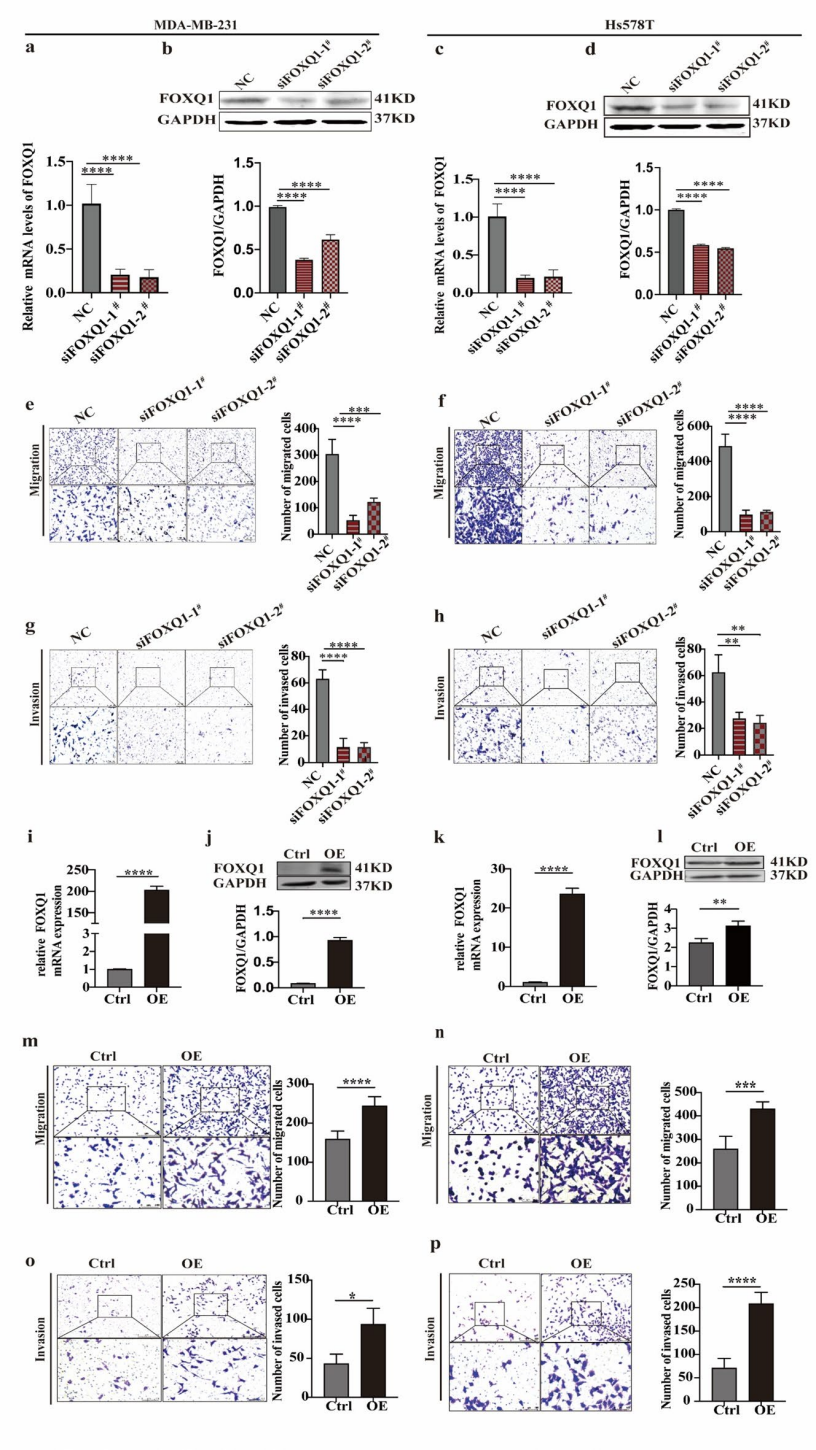
FOXQ1 plays a crucial role in enhancing the migration and invasion capabilities of TNBC cells. (**a**) The mRNA levels of FOXQ1 in MDA-MB-231 cells. MDA-MB-231 cells were transfected with either a negative control, siFOXQ1-1#, or siFOXQ1-2# for 24 h. The mRNA levels of FOXQ1 were measured using the qPCR method. (**b**) The protein levels of FOXQ1 in MDA-MB-231 cells. Cell processing is the same as a. Western blot measured the protein levels of FOXQ1. GAPDH serves as an internal control. (**c,d**) The mRNA (**c**) and protein (**d**) levels of FOXQ1 in Hs578T cells. (**e,f**) The migration capacity of MDA-MB-231 (**e**) and Hs578T cells (**f**). Cells were seeded onto a polycarbonate membrane and cultured in serum-free medium in the upper chamber, while the lower chamber was filled with culture medium supplemented with 20% serum to establish a chemotactic gradient. After 24 h of incubation, the number of cells that had migrated to the lower surface was quantified. (**g,h**) The invasion capacity of MDA-MB-231 (**g**) and Hs578T cells (**h**). The polycarbonate membrane was pre-coated with Matrigel, and the remaining procedures were identical to those in the previous migration assay. (**i,j**) qPCR and Western blot method verified FOXQ1’s mRNA (**i**) and protein (**j**) expression levels in MDA-MB-231 cells. (**k-l**) qPCR and Western blot verified FOXQ1’s mRNA (**k**) and protein (**l**) expression levels in Hs578T cells. Ctrl and OE represent a negative control and overexpressing FOXQ1, respectively. (**m,n**) The migration (**m**) and invasion (**n**) capability of control- and FOXQ1-overexpression MDA-MB-231 cells. (**o,p**) The migration (**o**) and invasion (**p**) capability of control- and FOXQ1-overexpression Hs578T cells. Statistical results were derived from three independent experiments and are presented as mean ± standard deviation (SD). **, ***, and **** indicate statistically significant differences with *p* values < 0.01, 0.001, and 0.0001, respectively, as determined by one-way ANOVA followed by Dunnett’s multiple comparisons test for three or more groups or the student’s t-test for two groups.

Having previously demonstrated that elevated FOXQ1 levels promote breast cancer cell proliferation and growth^10^, we further explored the functional role of FOXQ1 in TNBC progression. To evaluate its influence on cell migration and invasion, we selected two TNBC cell lines—MDA-MB-231 and Hs578T—that exhibit relatively high endogenous FOXQ1 expression. Efficient depletion of FOXQ1 mRNA was achieved by transfecting cells with two distinct siRNAs (si-1# and si-2#), as validated by qRT-PCR and immunoblotting (Fig. 2a and d). Knockdown of FOXQ1 significantly impaired the migratory (Fig. 2e and f) and invasive (Fig. 2g and h) abilities of both cell lines. In contrast, ectopic overexpression of FOXQ1 (Fig. 2i and l) markedly enhanced migration (Fig. 2m and n) and invasion (Fig. 2o and p) in MDA-MB-231 and Hs578T cells. Collectively, these findings demonstrate that FOXQ1 plays a critical role in driving the migratory and invasive phenotypes of TNBC cells.

### FOXQ1 enhances activation of the Wnt/β-catenin signaling pathway by upregulating WNT2 expression

**Fig. 3:**
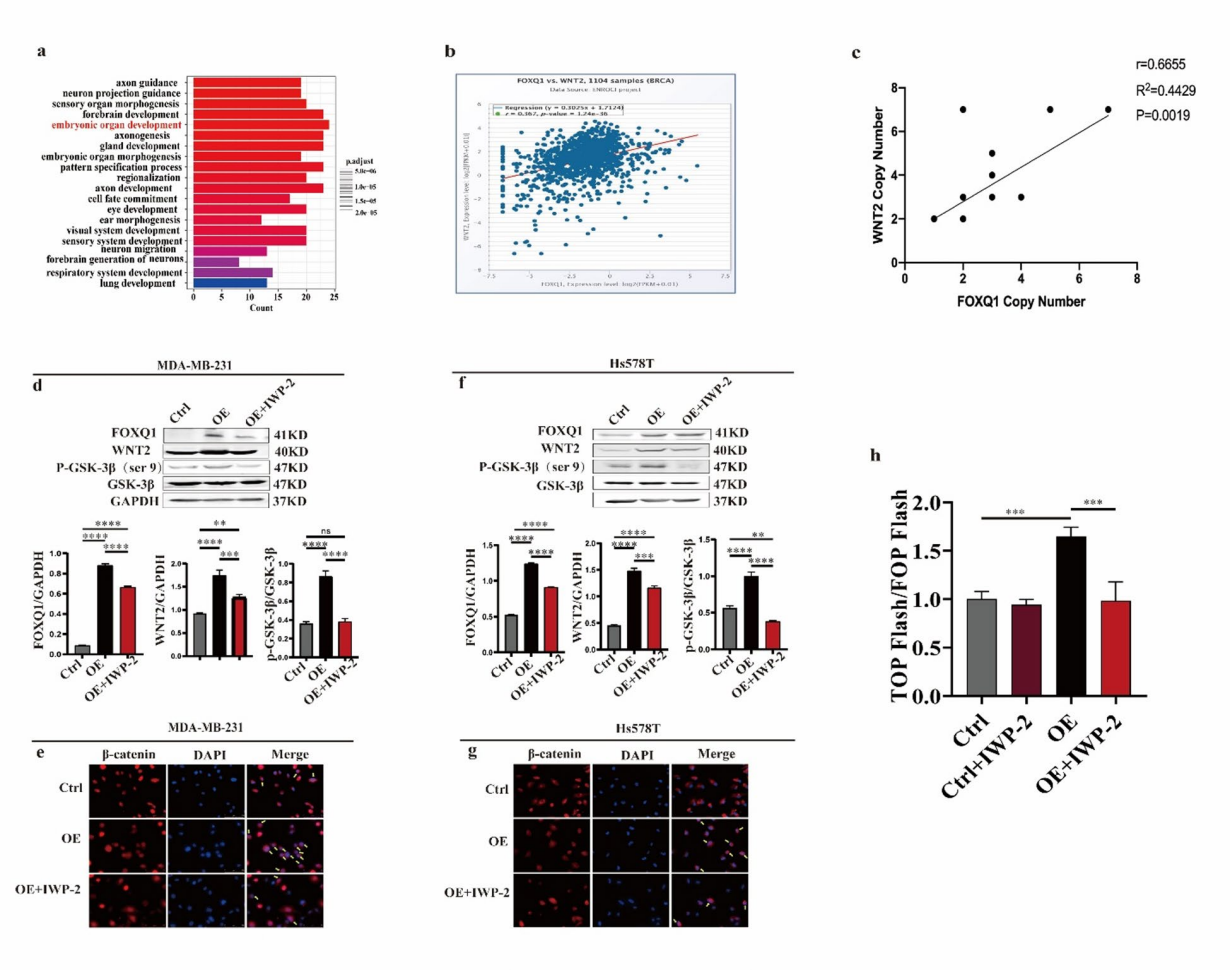
FOXQ1 enhanced Wnt/β-catenin signaling pathway activation by upregulating WNT2 expression. (**a**) Gene Ontology (GO) enrichment analysis of the target genes of FOXQ1. (**b,c**) Pearson correlation analysis of FOXQ1 and WNT2 mRNA levels. Data of 1104 breast cancer samples sourced from the Starbase database (**b**) and data of ten types of TNBC sourced from the Cancer Cell Line Encyclopedia (CCLE) database (**c**). (**d**) Protein levels of FOXQ1 and several vital molecules of the Wnt signaling pathway within MDA-MB-231 cells. Control and FOXQ1-overexpressing cells were treated with vehicle or IWP-2 (8 µM) for 24 h, and a Western blot was used to measure protein levels. (**e**) Immunofluorescence stain of β-catenin in MDA-MB-231. (**f**) Protein levels of FOXQ1 and several vital molecules of the Wnt signaling pathway within Hs578T cells. Cells were treated the same as (**d**). (**g**) Immunofluorescence stain of β-catenin in Hs578T. (**h**) Relative luciferase activity of TOP/FOP flash. HEK293T cells were transfected with either the TOP-Flash or FOP-Flash plasmid, together with a Renilla luciferase reporter plasmid for normalization. Following a 24-hour treatment with vehicle control, vehicle control plus IWP-2, FOXQ1-overexpression, or FOXQ1-overexpression plus IWP-2, firefly luciferase activity was measured and normalized to Renilla luciferase activity to correct for variations in transfection efficiency and cell viability. The FOP-Flash construct was used as a negative control. Statistical results were derived from three independent experiments and are presented as mean ± standard deviation (SD). Differences between groups were analyzed using Tukey’s multiple comparisons in One-way ANOVA. **, ***, **** and ns denote *P* < 0.01, 0.001, 0.0001 and non-significant.

To elucidate the molecular mechanism underlying the impact of FOXQ1 on TNBC migration and invasion, we conducted a bioinformatic analysis to predict the downstream targets of FOXQ1 by using the TRANSFAC (https://genexplain.com/transfac/) and TRRUST (https://www.grnpedia.org/trrust/) databases. We employed the overlap dataset derived from the two databases for GO enrichment analysis. FOXQ1 target genes are implicated in various biological processes, including embryonic organ development, axonogenesis, forebrain development, and gland development (Fig. 3a). Notably, the category of embryonic organ development was enriched with the highest number of genes (24 genes). Importantly, this category includes two key members of the Wnt family, WNT2 and WNT3A. We concentrated our subsequent investigation on the impact of FOXQ1 on WNT2 expression rather than on all WNT proteins. This decision was motivated by the critical role of WNT2 as a ligand for the frizzled receptor in initiating the canonical Wnt/β-catenin signaling pathway.

We initially examined the correlation between FOXQ1 and WNT2 expression. According to the Starbase database (https://starbase.sysu.edu.cn/), FOXQ1 and WNT2 expression levels had a positive correlation in 1104 breast cancer samples (*r* = 0.367, *p* < 0.05) (Fig. 3b). Furthermore, data from the Cancer Cell Line Encyclopedia (CCLE) database (https://sites.broadinstitute.org/ccle) also demonstrated a significant correlation between the copy numbers of these two genes (*r* = 0.6655, *p* = 0.0019) (Fig. 3c).

Subsequently, we investigated the impact of FOXQ1 on WNT2 protein expression and the activity of the Wnt/β-catenin signaling pathway. Consistent with our hypothesis, overexpression of FOXQ1 in MDA-MB-231 cells resulted in an approximately 1.8-fold increase in WNT2 expression, a two-fold increase in phosphorylation of GSK-3β (Fig. 3d), and enhanced nuclear localization of β-catenin (Fig. 3e). Notably, treatment with the WNT2 inhibitor IWP-2 abolished the FOXQ1-induced increase in GSK-3β phosphorylation and nuclear β-catenin localization without altering FOXQ1 overexpression-induced WNT2 upregulation (Fig. 3d and e). Similar results were observed in Hs578T cells (Fig. 3f and g). These findings suggest that FOXQ1 can upregulate WNT2 independently of Wnt/β-catenin signaling pathway activity.

Next, we examined the impact of FOXQ1 on the activity of the Wnt/β-catening pathway. The TOP/FOP Flash assay, a well-established method for assessing the activation of the Wnt/β-catenin signaling pathway, revealed that β-catenin-mediated transcriptional activity was nearly doubled in the FOXQ1-overexpressing cells compared with the control cells (Fig. 3h). Conversely when Wnt protein activity was inhibited by IWP-2, the overexpression of FOXQ1 no longer enhanced β-catenin-mediated transcriptional activity (Fig. 3h). These findings further confirmed that FOXQ1 enhances Wnt/β-catenin signaling pathway activity by upregulating WNT2 expression.

### WNT2 mediates EMT, migration, and invasion promoted by FOXQ1 in TNBC

**Fig. 4:**
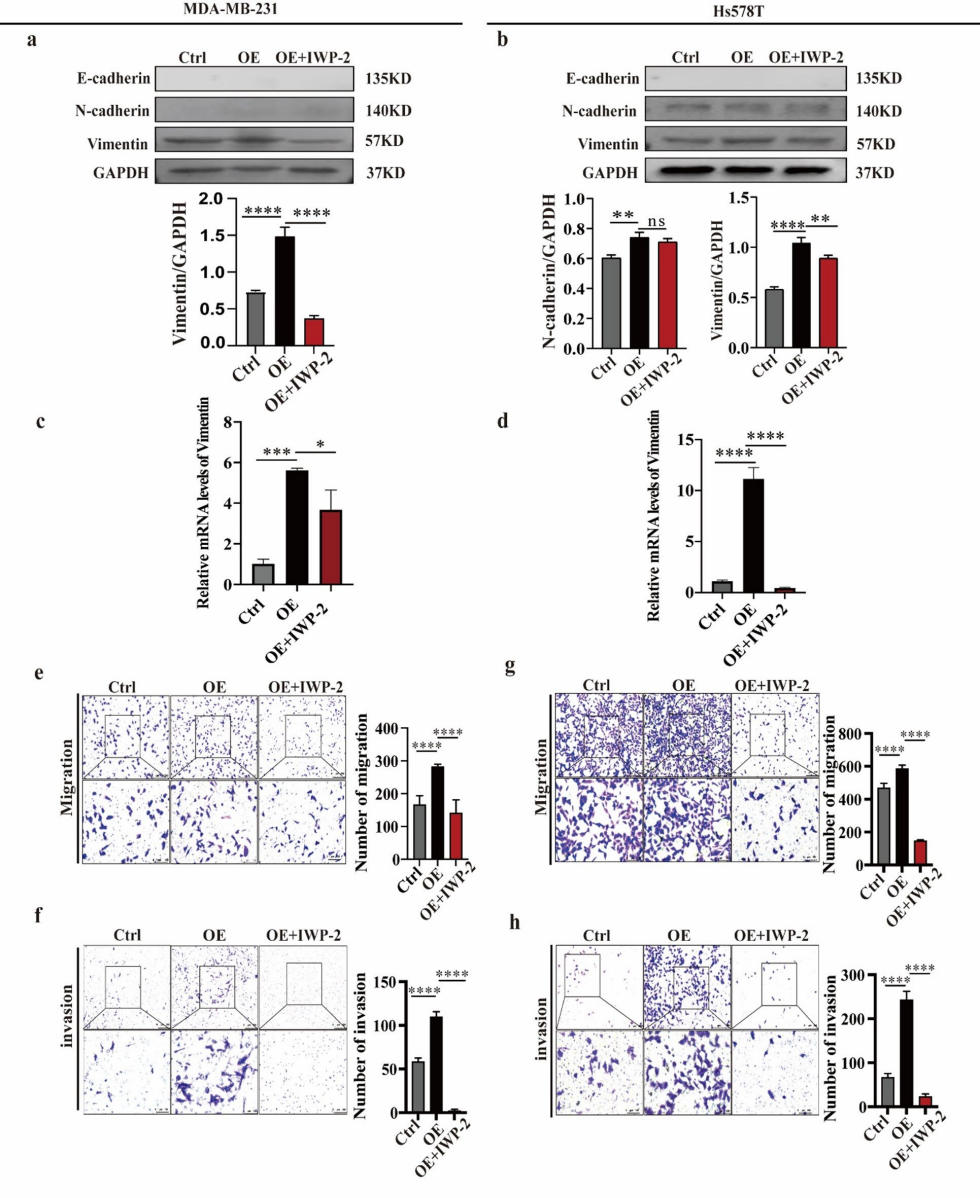
WNT2 mediates the EMT, migration, and invasion promoted by FOXQ1 in TNBC. (**a,b**) Protein levels of several EMT markers in MDA-MB-231 (**a**) and Hs578T cells (**b**). Control and FOXQ1-overexpressing cells were treated with vehicle or IWP-2 (8 µM) for 24 h. Western blot validated the protein levels. (**c,d**) Relative mRNA levels of Vimentin in MDA-MB-231 (**c**) and Hs578T cells (**d**). Control and FOXQ1-overexpressing cells were treated with vehicle or IWP-2 (8 µM) for 24 h. aPCR validated the mRNA levels. (**e,f**) The migration (**e**) and invasion (**f**) capabilities of MDA-MB-231. Control and FOXQ1-overexpressing cells were treated with the vehicle or IWP-2 (8 µM) for 24 h. The status of cell migration and invasion was examined using the transwell assay. (**g,h**) The migration (**g**) and invasion (**h**) capabilities of Hs578T cells. Cells were treated the same as (**e**) and (**f**). Statistical results were derived from three independent experiments and are presented as mean ± standard deviation (SD). Differences between groups were analyzed using Tukey’s multiple comparisons in one-way ANOVA. **, ****, and ns denote *P* < 0.01, 0.0001 and non-significant.

Subsequently, we investigated the role of WNT2 in FOXQ1-regulated migration and invasion of TNBC cells. Based on western blotting, MDA-MB-231 cells exhibited a lack of E-cadherin and N-cadherin protein expression. Overexpression of FOXQ1 resulted in elevated expression of vimentin, a marker of EMT. This effect was abrogated upon inhibition of WNT2 activity by IWP-2 treatment (Fig. 4a). We observed consistent results in Hs578T cells (Fig. 4b). A transwell assay further demonstrated that suppression of WNT2 by IWP-2 significantly diminished the migration and invasion of MDA-MB-231 (Fig. 4e and f) and Hs578T (Fig. 4g and h) cells. Collectively, these findings suggest that WNT2 mediates EMT, migration, and invasion, which FOXQ1 promotes in TNBC.

### miR-96-5p negatively regulates FOXQ1 expression by directly targeting the 3′UTR of its mRNA

**Fig. 5:**
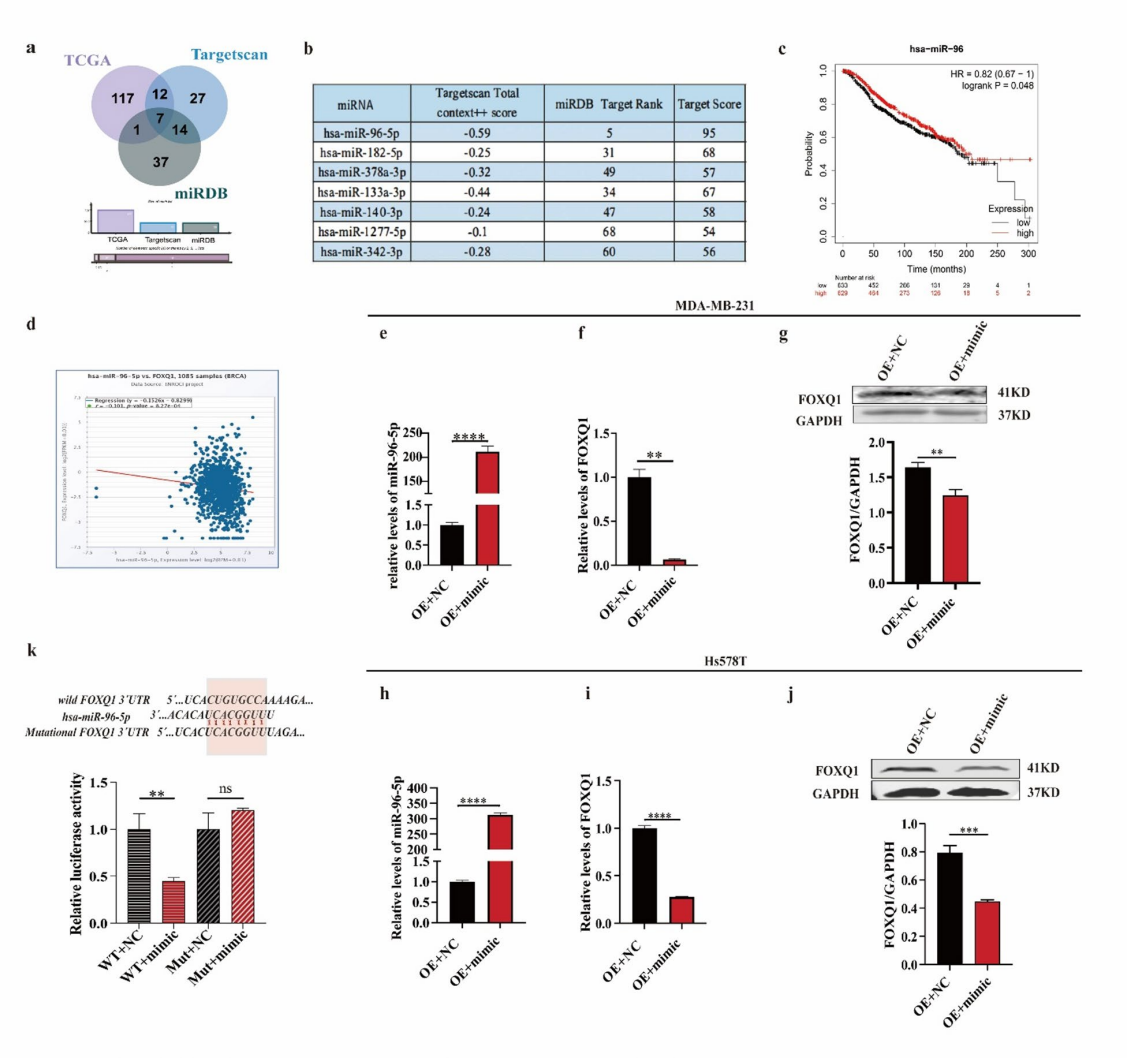
miR-96-5p negatively regulates FOXQ1 expression by directly targeting the 3’ UTR of its mRNA. (**a**) An overlap analysis of predicted miRNAs from three databases (TCGA, TargetScan, miRDB). (**b**) Overall score or rank of seven miRNAs. (**c**) The Kaplan-Meier plotter analysis revealed that patients with high miR-96-5p levels had better overall survival rates (*n* = 3033). All data were sourced from https://kmplot.com/analysis/. (**d**) Pearson correlation analysis of the mRNA levels of FOXQ1 and miR-96-5p within 1085 breast cancer samples. Data sourced from the Cancer Genome Atlas (TCGA) database. (**e,f**) Relative levels of miR-96-5p (**e**) and FOXQ1’s mRNA (**f**) in MDA-MB-231. FOXQ1-overexpression MDA-MB-231 cells were transfected with miR-96-5p mimic (mimic) or a negative control (NC). miR-96-5p and FOXQ1’s mRNA levels were measured using the qPCR method. (**g**) Protein levels of FOXQ1. FOXQ1-overexpression MDA-MB-231 cells were transfected with miR-96-5p mimics or a negative control for 24 h. Western blot validated the protein levels of FOXQ1. (**h-j**) Relative levels of miR-96-5p (**h**) and FOXQ1 (**i**), and protein levels of FOXQ1 (**j**) were measured in the same way as (**e-g**). method verification of the effect of miR-96-5p on FOXQ1’s mRNA in Hs578T cells. (**k**) Dual-luciferase reporter gene assay validated the binding site and role of miR-96-5p to FOXQ1. HEK-293T cells were transfected with either wild-type or mutant FOXQ1 3’UTR reporter plasmids. After 24 h of treatment with NC or miRNA mimic, relative luciferase activity was assessed using the Dual-Luciferase Reporter Assay system. Statistical results were derived from three independent experiments and are presented as the mean ± standard deviation (SD). Differences between groups were analyzed using unpaired T-tests. **, ***, ****, and ns present *P* < 0.01, 0.001, 0.0001, and no significance.

To identify specific endogenous molecules that inhibit FOXQ1, we performed a comprehensive analysis leveraging population and bioinformatics data. Initially, we screened for differentially expressed miRNAs in cancerous versus non-cancerous tissues from the TCGA database, identifying 137 miRNAs with at least a twofold change in expression (Fig. 5a). Then, we utilized the Targetscan (https://www.targetscan.org/vert_80/) and miRDB (https://mirdb.org/) databases to predict potential miRNAs that target FOXQ1, which yielded 69 and 59 candidates, respectively (Fig. 5a). By cross-referencing these datasets, we identified seven miRNAs present across all three sources: miR-96-5p, miR-182-5p, miR-378a-3p, miR-133a-3p, miR-140-3p, miR-1277-5p, and miR-342-3p (Fig. 5a and b). Among these, we selected miR-96-5p due to its high overall score (Fig. 5b) and its established role in regulating breast cancer growth, migration^24^, and invasion^25^.

We first validated the association between miR-96-5p expression and patient outcomes by using population data from the TCGA database. Kaplan-Meier survival analysis (https://kmplot.com/analysis/) revealed that patients with higher miR-96-5p expression exhibited significantly improved overall survival compared with those with lower miR-96-5p expression (HR = 0.82, *P* < 0.05) (Fig. 5c).

We then investigated the association between miR-96-5p levels and FOXQ1 expression by using data from the TCGA database. Pearson correlation analysis revealed a negative correlation between FOXQ1 mRNA expression and miR-96-5p expression in 1,085 breast cancer samples from the TCGA database (*r* = -0.101, *p* < 0.001) (Fig. 5d).

Based on the findings above, we investigated how miR-96-5p regulates the expression of FOXQ1. We increased miR-96-5p levels in FOXQ1-overexpressing MDA-MB-231 cells by transfecting them with miR-96-5p mimics (Fig. 5e). As a result, the FOXQ1 mRNA (Fig. 5f) and protein levels (Fig. 5g) were reduced to 10% and 70% of the control levels, respectively. Similarly, in FOXQ1-overexpressing Hs578T cells transfected with miR-96-5p mimics (Fig. 5h), the upregulation of miR-96-5p resulted in a decrease in FOXQ1 mRNA (Fig. 5i) and protein expression (Fig. 5j) to 20% and 50%, respectively. A dual luciferase reporter assay demonstrated that treatment with miR-96-5p mimics led to a more than 2-fold reduction in fluorescence intensity of the luciferase construct containing the wild-type FOXQ1 3′UTR compared with the control group (Fig. 5k). In contrast, we observed no significant effect for the construct harboring the mutant-type FOXQ1 3′UTR (Fig. 5k). These results robustly demonstrated that miR-96-5p specifically inhibits FOXQ1 expression by targeting its 3′UTR.

### miR-96-5p suppresses FOXQ1-mediated migration and invasion of TNBC

**Fig. 6:**
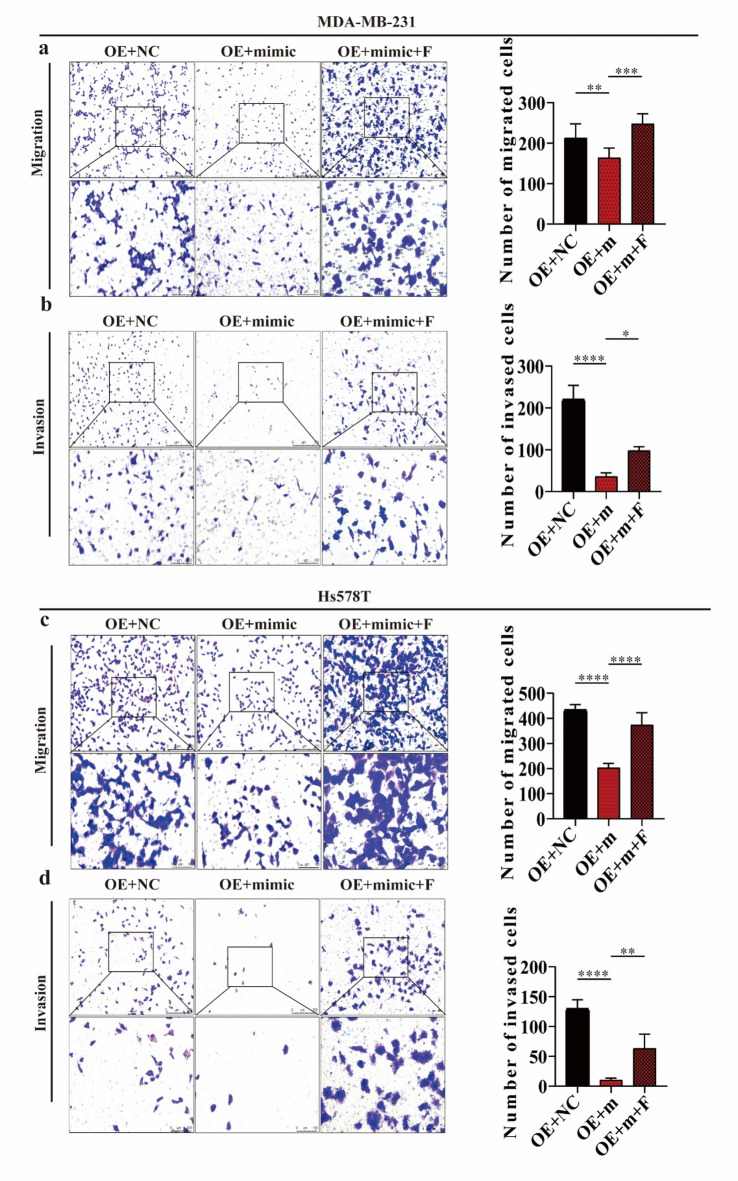
miR-96-5p suppresses FOXQ1-mediated migration and invasion of TNBC. (**a,b**) Transwell assay assessed the capabilities of migration (**a**) and invasion (**b**) of MDA-MB-231cells. MDA-MB-231 cells overexpressing FOXQ1 were transfected with miR-96-5p mimic (mimic), negative control (NC), or mimic co-transfected with FOXQ1 for 24 h prior to seeding into the upper chambers. Migration and invasion assays were performed, and the number of migrated or invaded cells was quantified after 24 h. (**c,d**) Transwell assay assessed the capabilities of migration (**a**) and invasion (**b**) of Hs578T cells. Cells are processed the same as (**a,d**). Statistical results were derived from three independent experiments and are presented as mean ± standard deviation (SD). Differences between groups were analyzed using one-way ANOVA or Student’s t-test. *, **, ***, **** and ns denote *P* < 0.05, 0.01, 0.001, 0.0001 and non-significant.

The introduction of miR-96-5p into FOXQ1-overexpressing MDA-MB-231 cells significantly inhibited cell migration and invasion (Fig. 6a and b). However, these inhibitory effects were reversed upon restoration of FOXQ1 expression (Fig. 6a and b). Similar results were observed in Hs578T cells (Fig. 6c and d). Collectively, these findings indicate that miR-96-5p suppresses TNBC cell migration and invasion by targeting FOXQ1.

### miR-96-5p inhibits FOXQ1-mediated malignant progression of TNBC in vivo

**Fig. 7:**
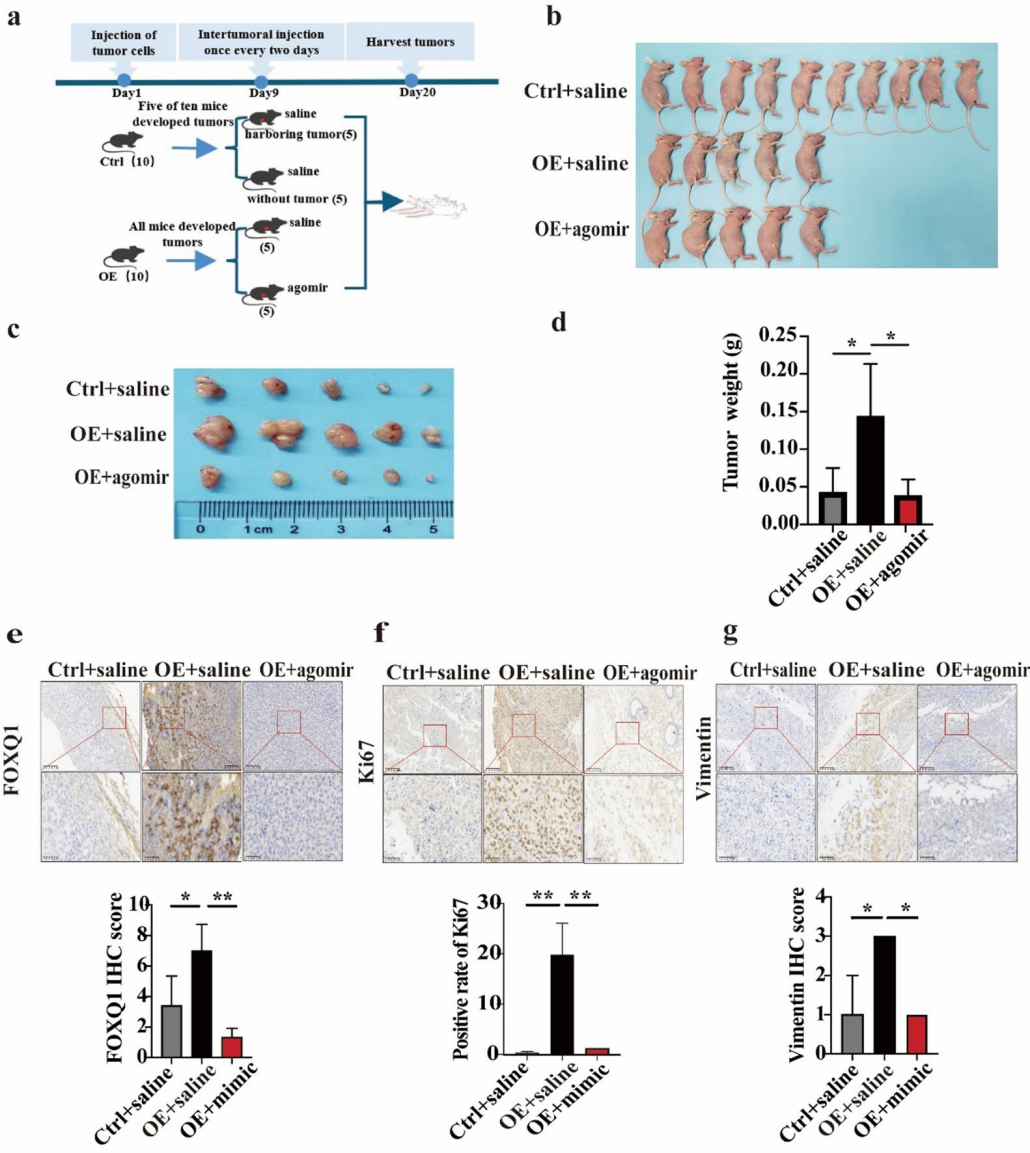
miR-96-5p inhibits breast cancer progression via FOXQ1 in vivo. (**a**) A flow chart of animal experiments. (**b**) Tumor-bearing status of BALB/c nude mice subjected to different treatments. (**c,d**) The tumor volume (**c**) and weight (**d**). (**e-g**) Immunuchemistrate strain for FOXQ1 (**e**), ki67 (**f**), and Vimentin (**g**) within MDA-MB-231 cells-drived tumor tissues. Tumors excised from mice were fixed in paraffin and sectioned for immunohistochemical analysis to evaluate the expression levels of FOXQ1, Ki67, and Vimentin in tumor tissues. Statistical results were derived from three independent experiments and are presented as mean ± standard deviation (SD). Differences between groups were analyzed using one-way ANOVA. * and ** denote *P* < 0.05 and 0.01.

We conducted animal experiments to investigate the in vivo impact of miR-96-5p on the malignant progression of TNBC. FOXQ1-overexpressing Hs578T cells and control cells were inoculated into the breast fat pad of BALB/C-nu mice, with 10 mice per group. Nine days post-inoculation, 50% of the mice (5 out of 10) in the control group had developed tumors. In contrast, all mice (10 out of 10) in the FOXQ1-overexpressed group developed tumors, resulting in a 100% tumorigenicity rate. These results provide robust evidence that overexpression of FOXQ1 promotes tumorigenesis in this experimental model (Fig. 7a).

Subsequently, we randomly and equally divided these mice harboring FOXQ1-overexpressing tumors into two groups: the saline control group (OE + saline) and the miR-96-5p agomir treatment group (OE + agomir), with five mice in each group. Meanwhile, the mice injected with control cells continued to receive saline treatments (Fig. 7a). After five times of intratumoral injections, the OE group exhibited larger tumors (Fig. 7b), both in terms of size (Fig. 7c) and weight (Fig. 7d), compared with the control group. In contrast, the OE + agomir group demonstrated markedly smaller tumors compared with the OE group (Fig. 7c and d) but did not exhibit a significant difference compared with the control group (Fig. 7d). Immunohistochemistry revealed a substantial increase in FOXQ1 levels in the OE group, approximately double compared with the control group (Fig. 7e). Concurrently, the OE group exhibited a 20-fold increase in Ki67 positivity (Fig. 7f) and a 3-fold increase in vimentin expression compared with the control group (Fig. 7g). Notably, the introduction of miR-96-5p agomir markedly reduced FOXQ1 levels (Fig. 7e) and reversed the elevated Ki67 (Fig. 7f) and vimentin expression (Fig. 7g). These results suggest that FOXQ1 overexpression significantly enhances the growth and EMT of TNBC, while miR-96-5p can inhibit the FOXQ1-driven malignant progression of TNBC in vivo.

## Discussion

**Fig. 8:**
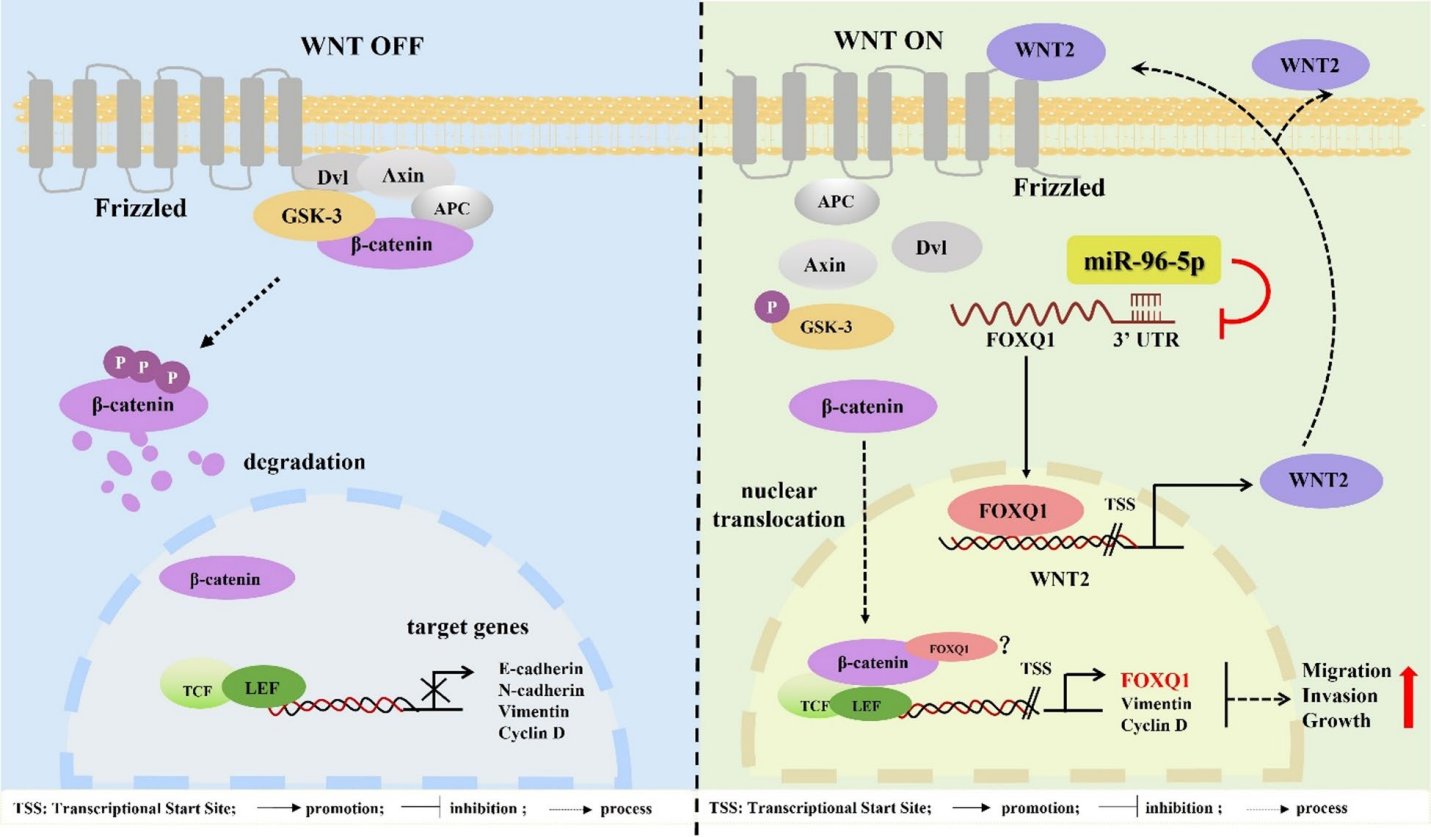
A schematic diagram of the miR-96-5p/FOXQ1/WNT2/β-catenin regulatory axis in triple-negative breast cancer (TNBC) cells. FOXQ1 directly upregulates WNT2, a potent ligand of the Wnt/β-catenin signaling pathway, leading to enhanced phosphorylation of GSK-3β and subsequent nuclear translocation of β-catenin. This activation promotes the expression of downstream Wnt target genes, including FOXQ1 itself, forming a positive feedback loop that drives tumor migration, invasion, and growth in triple-negative breast cancer (TNBC). However, miR-96-5p binds to the 3’UTR of FOXQ1 mRNA, suppressing its expression. As a result, this inhibition disrupts the Wnt/β-catenin signaling cascade and attenuates TNBC progression.

Triple-negative breast cancer (TNBC) is characterized by aggressive clinical behavior, including rapid proliferation, epithelial–mesenchymal transition (EMT), and a high propensity for metastasis^3,26–28^. Although FOXQ1 has been linked to the activation of Wnt/β-catenin signaling in cancer progression, the precise molecular mechanisms underlying this relationship remain incompletely understood. In this study, we demonstrate that FOXQ1 enhances Wnt/β-catenin signaling by upregulating the Wnt ligand WNT2, thereby promoting the aggressiveness of TNBC. Our findings reveal a previously unrecognized regulatory axis between FOXQ1 and WNT2 that drives Wnt pathway activation, providing new mechanistic insights into the progression of TNBC (Fig. 8).

The canonical Wnt/β-catenin pathway, a key regulator of cell fate and EMT, is activated by Wnt ligands that stabilize β-catenin, enabling its nuclear translocation to drive the expression of pro-tumorigenic genes like SNAIL and c-MYC^29,30^. Among these ligands, WNT2 acts as a key activator of the canonical pathway. Although essential in embryonic development, WNT2 is often silenced in adult tissues but is reactivated in multiple malignancies^31–33^. For instance, in gastric cancer, WNT2 cooperates with SOX4 to form a positive feedback loop that sustains the self-renewal of cancer stem cells^34^. In oesophageal cancer, WNT2 promotes cancer cell proliferation by activating the Wnt/β-catenin signalling pathway, leading to the upregulation of cyclin D1 and c-myc expression^32^. Thus, WNT2 functions not merely as a binary Wnt switch but as a dynamic signaling hub embedded within broader oncogenic networks^35^.

Previous studies, including one on colorectal cancer, have identified FOXQ1 as a transcriptional target of the Wnt/β-catenin signaling pathway^36^. Here, we uncover a reverse regulatory relationship in TNBC: bioinformatic analysis revealed a conserved FOXQ1-binding motif in the WNT2 promoter, and functional studies confirmed that FOXQ1 overexpression upregulates WNT2 expression, stimulates β-catenin nuclear translocation, and enhances downstream transcriptional activity. Critically, inhibition of Wnt ligand palmitoylation and secretion using IWP-2 abolished β-catenin activation but did not suppress FOXQ1-induced WNT2 upregulation, indicating that FOXQ1 transcriptionally controls WNT2 independently of canonical Wnt/β-catenin signaling. Interestingly, IWP-2 treatment partially reduced FOXQ1 protein levels, suggesting that FOXQ1 expression remains influenced by Wnt activity, which is consistent with earlier reports in colorectal cancer^37^. Together, these data support a model in which FOXQ1, WNT2, and β-catenin form a positive feedback loop that sustains constitutive Wnt pathway activation in TNBC. This self-reinforcing circuit may underlie the persistent aggressiveness of this breast cancer subtype (Fig. 8).

Based on the established role of this pathogenic feedback loop, we further explored its therapeutic potential. We identified miR-96-5p as a microRNA that directly targets FOXQ1 and effectively suppresses its expression. Although miR-96-5p is known for its context-dependent oncogenic or tumor-suppressive roles across various cancers^38–41^, our data firmly establish its tumor-suppressive function in TNBC. Analysis of TCGA data revealed that high expression of miR-96-5p is significantly associated with improved survival in a cohort of 3,033 breast cancer patients, consistent with a previous report that it suppresses breast cancer metastasis through CTNND1^42^. Mechanistically, we confirmed that miR-96-5p directly binds to the 3’UTR of FOXQ1, leading to the suppression of FOXQ1 expression and subsequent inhibition of downstream Wnt/β‑catenin signaling. This interaction was shown to be specific, as mutating the miR-96-5p binding site abolished its regulatory effect. Functionally, overexpression of miR-96-5p effectively counteracted FOXQ1-driven proliferation, migration, and invasion in both in vitro and in vivo models, underscoring its therapeutic promise.

Collectively, our work delineates a previously unrecognized pathogenic circuit in TNBC, wherein a positive feedback loop connecting FOXQ1, WNT2, and β-catenin drives and sustains Wnt pathway hyperactivation. The identification of miR-96-5p as a direct and potent suppressor of FOXQ1 not only provides mechanistic insight but also unveils a promising therapeutic vulnerability.

Several considerations for future work emerge from our findings. First, while our data firmly establish the core FOXQ1-WNT2-β-catenin axis, the potential involvement of additional parallel effectors downstream of FOXQ1 remains to be fully elucidated. Second, the generalizability of this signaling module across the spectrum of TNBC heterogeneity, beyond the mesenchymal-subtype models primarily used here, awaits validation in a broader panel of models, including basal-like subtypes and patient-derived xenografts. Addressing these questions will be crucial for defining the patient population most likely to benefit from this strategy.

Notwithstanding these limitations, the therapeutic implications are compelling. The efficacy of miR-96-5p in suppressing FOXQ1-driven oncogenicity, coupled with the maturity of oligonucleotide delivery platforms such as lipid nanoparticles, positions the restoration of miR-96-5p function as a viable and attractive strategy. Future efforts should focus on advancing delivery efficacy and exploring rational combination regimens, such as those with Wnt inhibitors, to achieve durable pathway suppression and circumvent potential resistance. Ultimately, targeting the FOXQ1-miR-96-5p node offers a novel avenue to disrupt a key self-reinforcing signaling circuit in TNBC.

##  Conclusion

In conclusion, we define a self-reinforcing FOXQ1-WNT2-β-catenin feedback loop as a key driver of Wnt pathway hyperactivation in TNBC, and identify its potent suppression by miR-96-5p as a promising therapeutic strategy.

## Supplementary Information

Below is the link to the electronic supplementary material.

Supplementary Material 1

Supplementary Material 2

## Data Availability

The datasets used and/or analyzed during the current study are available from the corresponding author upon reasonable request.

## Abbreviations

TNBC: Triple-negative breast cancer
EMT: Epithelial–mesenchymal transition
Wnt: Wingless/integrated
miR: MicroRNA
RNA: Ribonucleic acid
GSK-3β: Glycogen synthase kinase 3 beta
Dvl: Dishevelled
mRNA: Messenger-ribonucleic acid
3’UTR 3’: Untranslated region
GAPDH: Glyceraldehyde-3-phosphate dehydrogenase
PBS: Phosphate buffer saline
GO: Gene ontology

## References

[1] Bray, F. et al. Global cancer statistics 2022: GLOBOCAN estimates of incidence and mortality worldwide for 36 cancers in 185 countries. *CA Cancer J. Clin.*10.3322/caac.21834 (2024). 38572751 10.3322/caac.21834

[2] Guestini, F., McNamara, K. M., Ishida, T. & Sasano, H. Triple negative breast cancer chemosensitivity and chemoresistance: Current advances in biomarkers indentification. *Expert Opin. Ther. Targets*. **20**, 705–720. 10.1517/14728222.2016.1125469 (2016). 26607563 10.1517/14728222.2016.1125469

[3] Singh, D. D. & Yadav, D. K. TNBC: Potential targeting of multiple receptors for a therapeutic breakthrough, nanomedicine, and immunotherapy. *Biomedicines* **9**. 10.3390/biomedicines9080876 (2021). 10.3390/biomedicines9080876 PMC8389539 34440080

[4] Gluz, O. et al. Triple-negative breast cancer–current status and future directions. *Ann. Oncol.* **20**, 1913–1927. 10.1093/annonc/mdp492 (2009). 19901010 10.1093/annonc/mdp492

[5] Bieller, A. et al. Isolation and characterization of the human forkhead gene FOXQ1. *DNA Cell Biol*. **20** 555–561 (2001). 10.1089/104454901317094963 10.1089/104454901317094963 11747606

[6] Tang, H., Zhang, J. & Guo, Q. Research progress on the regulation of tumor initiation and development by the forkhead box Q1 gene. *J. Cancer Res. Ther.* **14**, 6–11. 10.4103/jcrt.JCRT_701_17 (2018). 29516951 10.4103/jcrt.JCRT_701_17

[7] Wu, C. et al. FOXQ1 promotes pancreatic cancer cell proliferation, tumor stemness, invasion and metastasis through regulation of LDHA-mediated aerobic glycolysis. *Cell. Death Dis.* **14**, 699. 10.1038/s41419-023-06207-y (2023). 37875474 10.1038/s41419-023-06207-y PMC10598070

[8] Mitchell, A. V. et al. FOXQ1 recruits the MLL complex to activate transcription of EMT and promote breast cancer metastasis. *Nat. Commun.* **13**, 6548. 10.1038/s41467-022-34239-z (2022). 36319643 10.1038/s41467-022-34239-z PMC9626503

[9] Wu, J. et al. PARP1-stabilised FOXQ1 promotes ovarian cancer progression by activating the LAMB3/WNT/β-catenin signalling pathway. *Oncogene* **43**, 866–883. 10.1038/s41388-024-02943-3 (2024). 38297082 10.1038/s41388-024-02943-3

[10] Lin, Y. et al. The FGFR1 signaling pathway upregulates the oncogenic transcription factor FOXQ1 to promote breast cancer cell growth. *Int. J. Biol. Sci.* **19**, 744–759. 10.7150/ijbs.74574 (2023). 36778115 10.7150/ijbs.74574 PMC9909991

[11] Liu, Q. et al. Nuclear isoform of RAPH1 interacts with FOXQ1 to promote aggressiveness and radioresistance in breast cancer. *Cell. Death Dis.* **14**, 803. 10.1038/s41419-023-06331-9 (2023). 38062011 10.1038/s41419-023-06331-9 PMC10703867

[12] Koch, S. The transcription factor FOXQ1 in cancer. *Cancer Metastasis Rev.* **44**, 22. 10.1007/s10555-025-10240-y (2025). 39777582 10.1007/s10555-025-10240-y PMC11711781

[13] Nusse, R. & Varmus, H. E. Many tumors induced by the mouse mammary tumor virus contain a provirus integrated in the same region of the host genome. *Cell* **31**, 99–109. 10.1016/0092-8674(82)90409-3 (1982). 6297757 10.1016/0092-8674(82)90409-3

[14] Nusse, R. & Clevers, H. Wnt/β-catenin signaling, disease, and emerging therapeutic modalities. *Cell* **169**, 985–999. 10.1016/j.cell.2017.05.016 (2017). 28575679 10.1016/j.cell.2017.05.016

[15] Majidinia, M., Aghazadeh, J., Jahanban-Esfahlani, R. & Yousefi, B. The roles of Wnt/β-catenin pathway in tissue development and regenerative medicine. *J. Cell. Physiol.* **233**, 5598–5612. 10.1002/jcp.26265 (2018). 29150936 10.1002/jcp.26265

[16] Liu, J. et al. Wnt/β-catenin signalling: Function, biological mechanisms, and therapeutic opportunities. *Signal. Transduct. Target. Ther.* **7**, 3. 10.1038/s41392-021-00762-6 (2022). 34980884 10.1038/s41392-021-00762-6 PMC8724284

[17] Zhu, L. et al. PROX1 promotes breast cancer invasion and metastasis through WNT/β-catenin pathway via interacting with HnRNPK. *Int. J. Biol. Sci.* **18**, 2032–2046. 10.7150/ijbs.68960 (2022). 35342346 10.7150/ijbs.68960 PMC8935233

[18] Lin, Y. et al. Effect of miR-133b on progression and cisplatin resistance of triple-negative breast cancer through FGFR1-Wnt-β-catenin axis. *Am. J. Transl Res.* **13**, 5969–5984 (2021). 34306338 PMC8290659

[19] Peng, X. et al. FOXQ1 mediates the crosstalk between TGF-beta and Wnt signaling pathways in the progression of colorectal cancer. *Cancer Biol. Ther.* **16**, 1099–1109. 10.1080/15384047.2015.1047568 (2015). 25955104 10.1080/15384047.2015.1047568 PMC4623466

[20] Lu, T. X. & Rothenberg, M. E. MicroRNA. *J. Allergy Clin. Immunol.* **141**, 1202–1207. 10.1016/j.jaci.2017.08.034 (2018). 10.1016/j.jaci.2017.08.034 PMC5889965 29074454

[21] Mansoori, B. et al. MiR-142-3p targets HMGA2 and suppresses breast cancer malignancy. *Life Sci.* **276**, 119431. 10.1016/j.lfs.2021.119431 (2021). 33785332 10.1016/j.lfs.2021.119431

[22] Xu, J. H. et al. MiR-193 promotes cell proliferation and invasion by ING5/PI3K/AKT pathway of triple-negative breast cancer. *Eur. Rev. Med. Pharmacol. Sci.* **24**, 3122–3129. 10.26355/eurrev_202003_20679 (2020). 32271430 10.26355/eurrev_202003_20679

[23] Chen, B. et al. Small molecule-mediated disruption of Wnt-dependent signaling in tissue regeneration and cancer. *Nat. Chem. Biol.* **5**, 100–107. 10.1038/nchembio.137 (2009). 19125156 10.1038/nchembio.137 PMC2628455

[24] Gao, Z. et al. Long non-coding RNA CASC2 inhibits breast cancer cell growth and metastasis through the regulation of the miR-96-5p/SYVN1 pathway. *Int. J. Oncol.* **53**, 2081–2090. 10.3892/ijo.2018.4522 (2018). 30106139 10.3892/ijo.2018.4522

[25] Gao, X. H. et al. MicroRNA-96-5p represses breast cancer proliferation and invasion through Wnt/beta-catenin signaling via targeting CTNND1. *Sci. Rep.* **10**, 44. 10.1038/s41598-019-56571-z (2020). 31913290 10.1038/s41598-019-56571-z PMC6949244

[26] Yin, L., Duan, J. J., Bian, X. W. & Yu, S. C. Triple-negative breast cancer molecular subtyping and treatment progress. *Breast Cancer Res.* **22**, 61. 10.1186/s13058-020-01296-5 (2020). 32517735 10.1186/s13058-020-01296-5 PMC7285581

[27] Deepak, K. G. K. et al. Tumor microenvironment: Challenges and opportunities in targeting metastasis of triple negative breast cancer. *Pharmacol. Res.* **153**, 104683. 10.1016/j.phrs.2020.104683 (2020). 32050092 10.1016/j.phrs.2020.104683

[28] Lyons, T. G. Targeted therapies for triple-negative breast cancer. *Curr. Treat. Options Oncol.* **20**, 82. 10.1007/s11864-019-0682-x (2019). 31754897 10.1007/s11864-019-0682-x

[29] Taciak, B., Pruszynska, I., Kiraga, L., Bialasek, M. & Krol, M. Wnt signaling pathway in development and cancer. *J. Physiol. Pharmacol.* **69**. 10.26402/jpp.2018.2.07 (2018). 10.26402/jpp.2018.2.07 29980141

[30] Clevers, H. Wnt/beta-catenin signaling in development and disease. *Cell* **127**, 469–480. 10.1016/j.cell.2006.10.018 (2006). 17081971 10.1016/j.cell.2006.10.018

[31] Jung, Y. S., Jun, S., Lee, S. H., Sharma, A. & Park, J. I. Wnt2 complements Wnt/β-catenin signaling in colorectal cancer. *Oncotarget* **6**, 37257–37268. 10.18632/oncotarget.6133 (2015). 26484565 10.18632/oncotarget.6133 PMC4741928

[32] Fu, L. et al. Wnt2 secreted by tumour fibroblasts promotes tumour progression in oesophageal cancer by activation of the Wnt/β-catenin signalling pathway. *Gut* **60**, 1635–1643. 10.1136/gut.2011.241638 (2011). 21672941 10.1136/gut.2011.241638

[33] Huang, T. X. et al. Targeting cancer-associated fibroblast-secreted WNT2 restores dendritic cell-mediated antitumour immunity. *Gut* **71**, 333–344. 10.1136/gutjnl-2020-322924 (2022). 33692094 10.1136/gutjnl-2020-322924 PMC8762012

[34] Tan, X. Y. et al. WNT2-SOX4 positive feedback loop promotes chemoresistance and tumorigenesis by inducing stem-cell like properties in gastric cancer. *Oncogene* **42**, 3062–3074. 10.1038/s41388-023-02816-1 (2023). 37634009 10.1038/s41388-023-02816-1

[35] Mentucci, F. M., Ferrara, M. G., Ercole, A., Rumie Vittar, N. B. & Lamberti, M. J. Interplay between cancer-associated fibroblasts and dendritic cells: implications for tumor immunity. *Front. Immunol.* **16**, 1515390. 10.3389/fimmu.2025.1515390 (2025). 40453074 10.3389/fimmu.2025.1515390 PMC12122520

[36] Christensen, J., Bentz, S., Sengstag, T., Shastri, V. P. & Anderle, P. FOXQ1, a novel target of the Wnt pathway and a new marker for activation of Wnt signaling in solid tumors. *PloS One*. **8**, e60051. 10.1371/journal.pone.0060051 (2013). 23555880 10.1371/journal.pone.0060051 PMC3608605

[37] Pizzolato, G., Moparthi, L., Söderholm, S., Cantù, C. & Koch, S. The oncogenic transcription factor FOXQ1 is a differential regulator of Wnt target genes. *J. Cell Sci.* **135**. 10.1242/jcs.260082 (2022). 10.1242/jcs.260082 36124643

[38] Iwai, N. et al. Oncogenic miR-96-5p inhibits apoptosis by targeting the caspase-9 gene in hepatocellular carcinoma. *Int. J. Oncol.* **53**, 237–245. 10.3892/ijo.2018.4369 (2018). 29658604 10.3892/ijo.2018.4369

[39] Wang, T., Xu, Y., Liu, X., Zeng, Y. & Liu, L. miR-96-5p is the tumor suppressor in osteosarcoma via targeting SYK. *Biochem. Biophys. Res. Commun.* **572**, 49–56. 10.1016/j.bbrc.2021.07.069 (2021). 34343834 10.1016/j.bbrc.2021.07.069

[40] Canovai, M. et al. miR-210-3p, miR-183-5p and miR-96-5p reduce sensitivity to docetaxel in prostate cancer cells. *Cell. Death Discov*. **9**, 445. 10.1038/s41420-023-01696-4 (2023). 38065937 10.1038/s41420-023-01696-4 PMC10709610

[41] Zhang, H., Chen, R. & Shao, J. MicroRNA-96-5p facilitates the viability, migration, and invasion and suppresses the apoptosis of cervical cancer cells by negatively modulating SFRP4. *Technol. Cancer Res. Treat.* **19**, 1533033820934132. 10.1177/1533033820934132 (2020). 32527205 10.1177/1533033820934132 PMC7294480

[42] Gao, X. H. et al. MicroRNA-96-5p represses breast cancer proliferation and invasion through Wnt/β-catenin signaling via targeting CTNND1. *Sci. Rep.* **10**, 44. 10.1038/s41598-019-56571-z (2020). 31913290 10.1038/s41598-019-56571-z PMC6949244

