# Supplementary material for: miR-96-5p antagonizes FOXQ1-driven WNT/β-catenin signaling to inhibit triple-negative breast cancer
Source: Sci Rep. 2026 Jan 4;16:4624. doi: 10.1038/s41598-025-34859-7 (PMC12868773; doi:10.1038/s41598-025-34859-7)

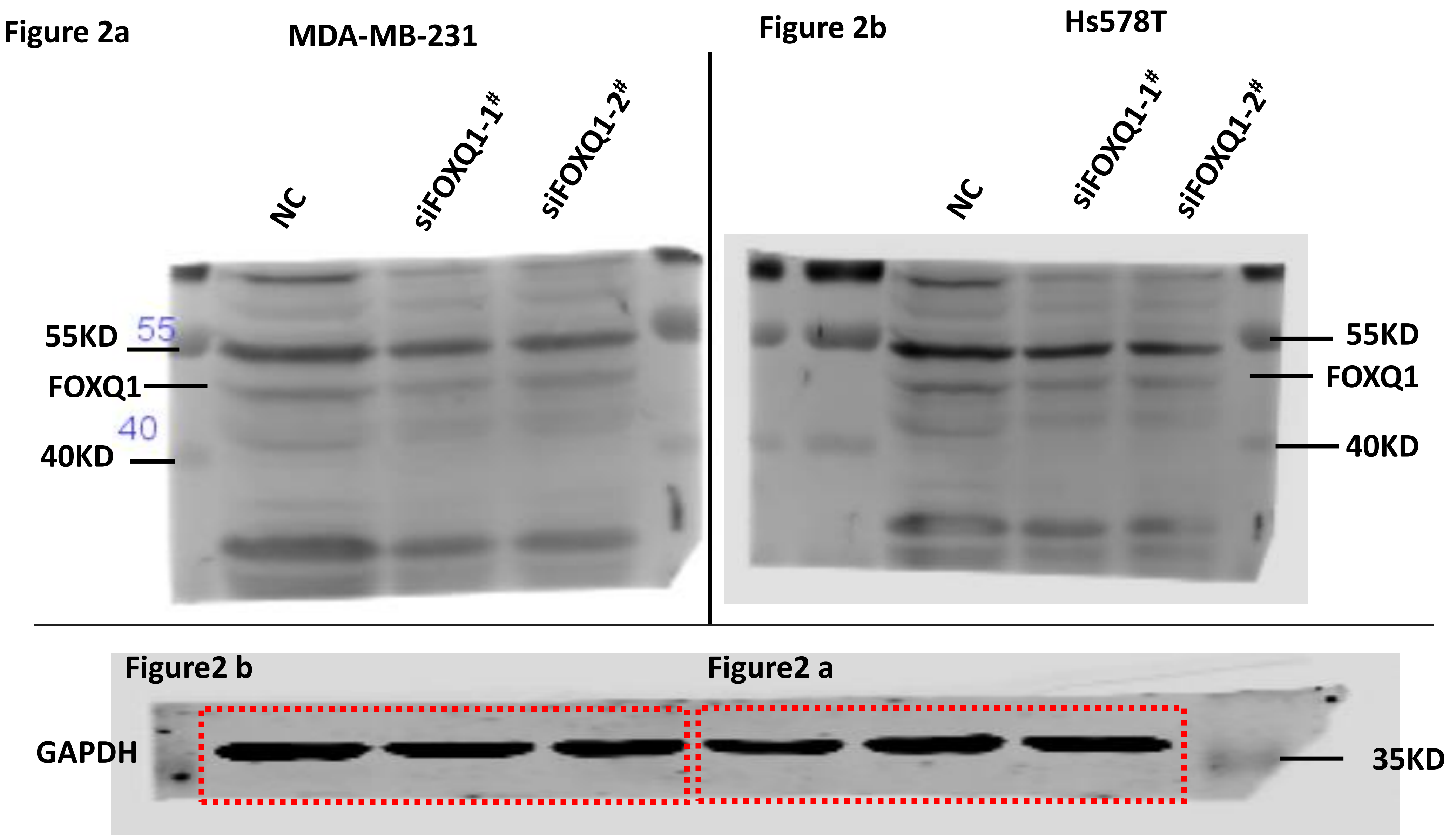


Lane 1-3: GAPDH of Hs578T (Fig.2b); Lane 4-6: GAPDH of MDA-MB-231(Fig. 2a)

Figure 2h MDA-MB -231

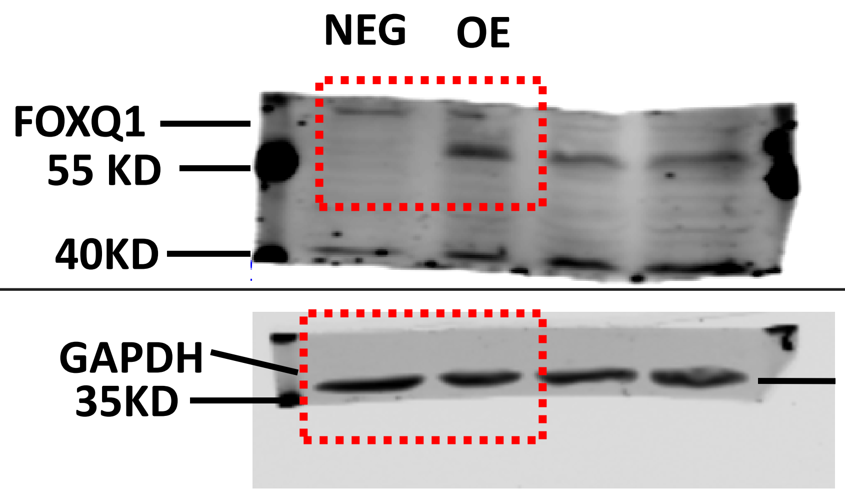


Figure 2j Hs578T

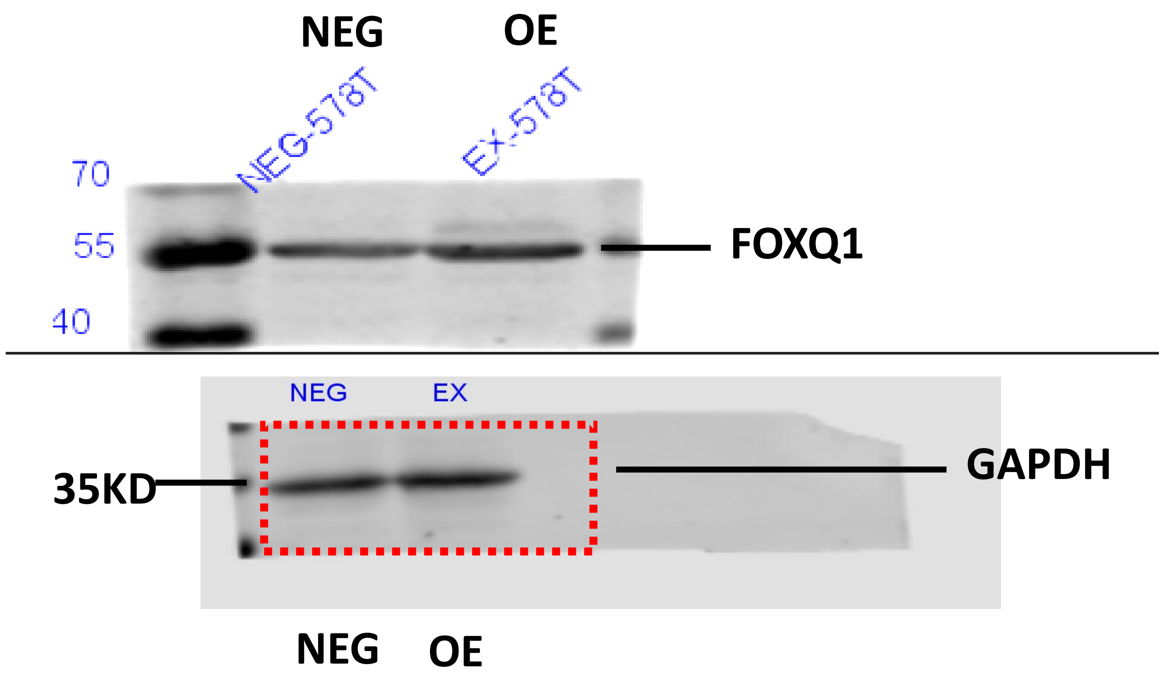

Figure 3d

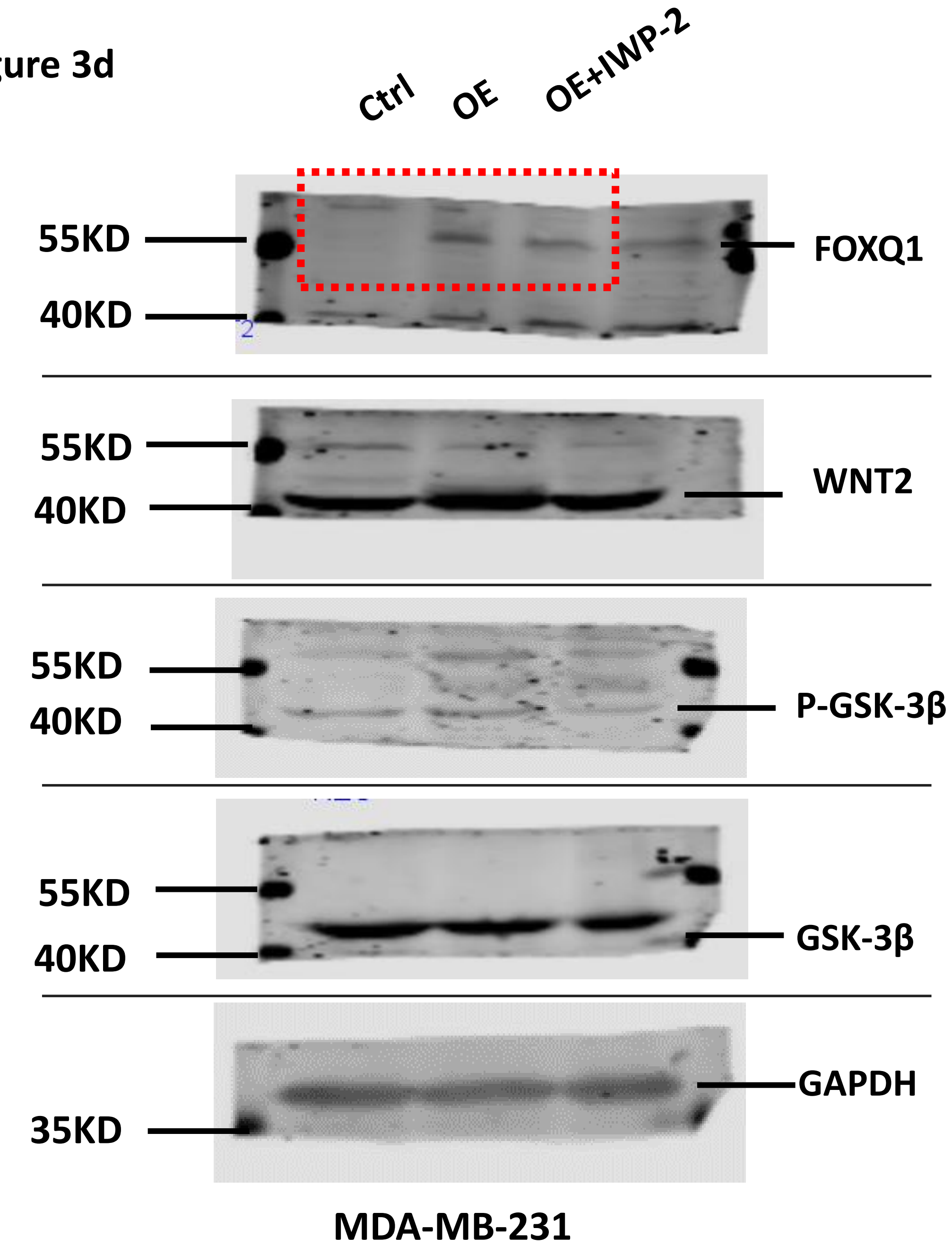


Figure 3f

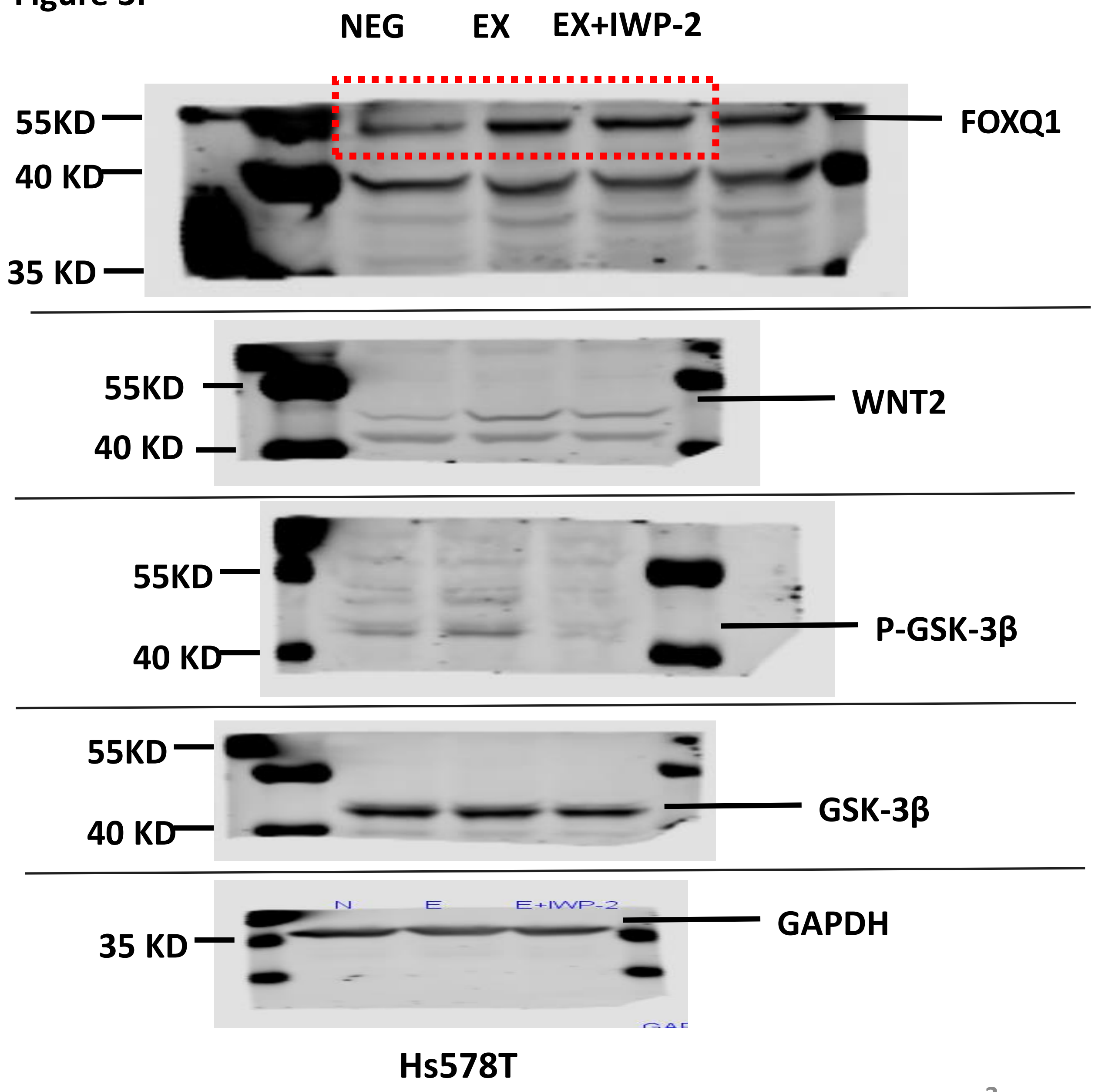

Figure 4a

MDA-MB-231

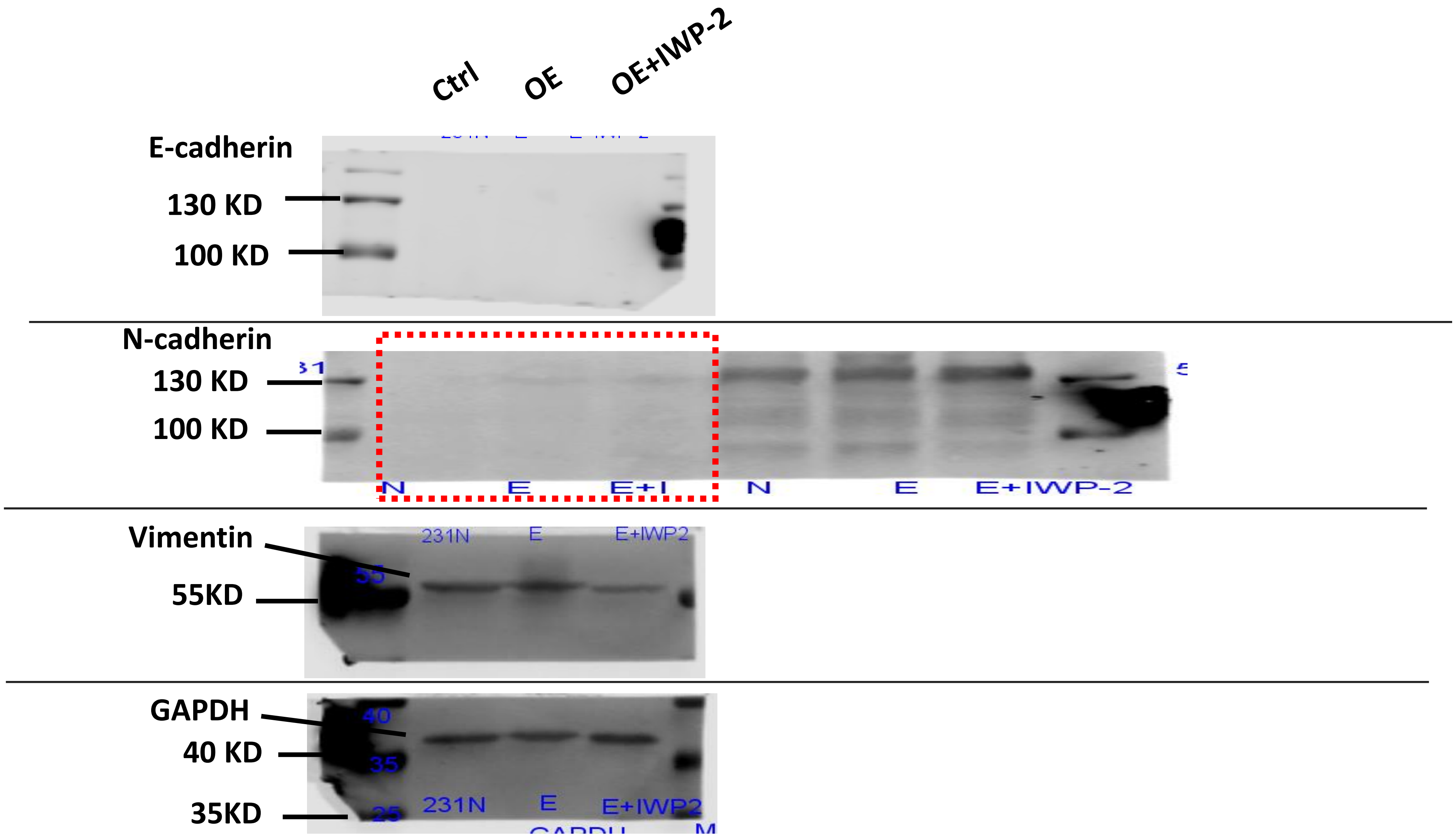

**Figure 4b** Hs578T

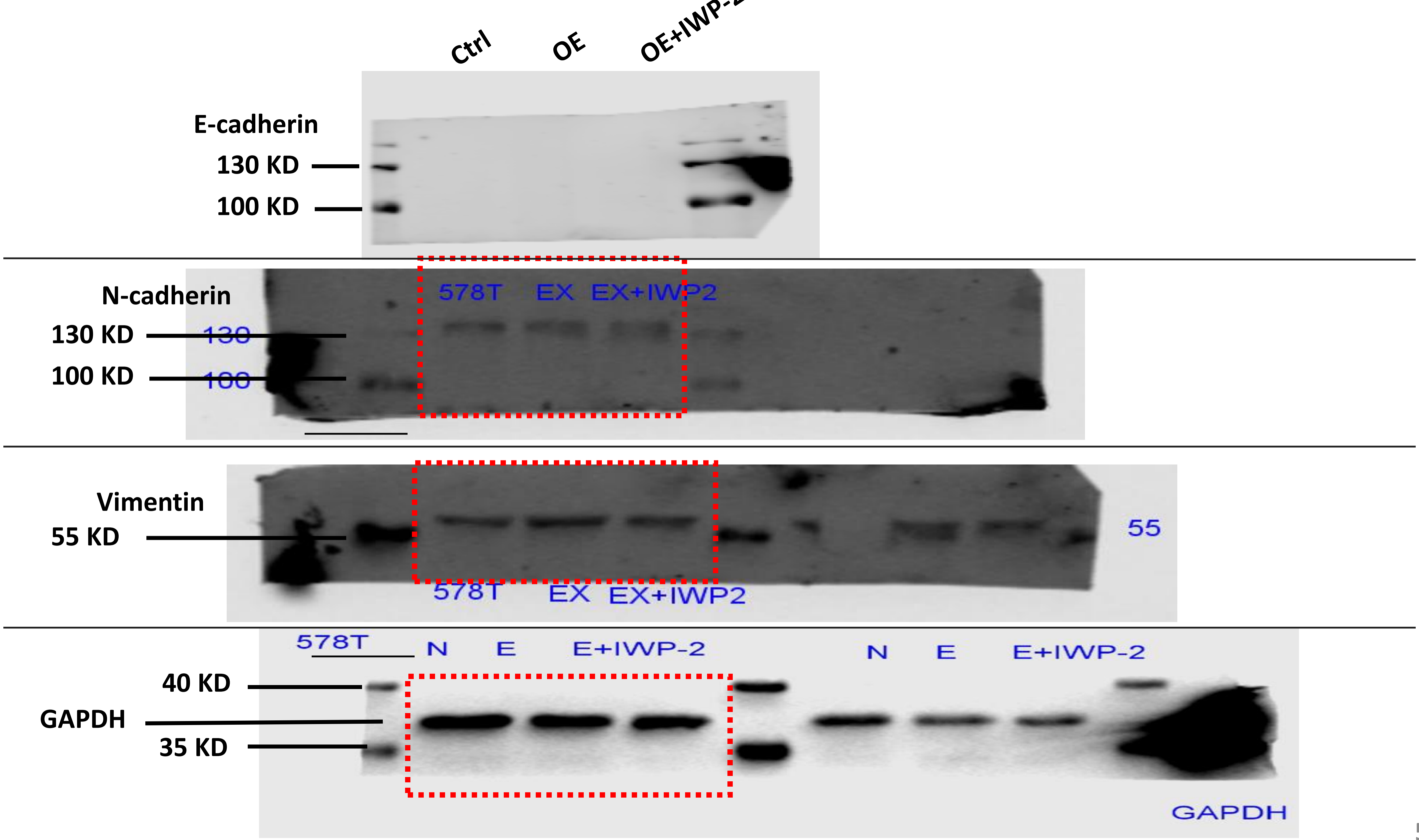

Figure 5f MDA-MB-231

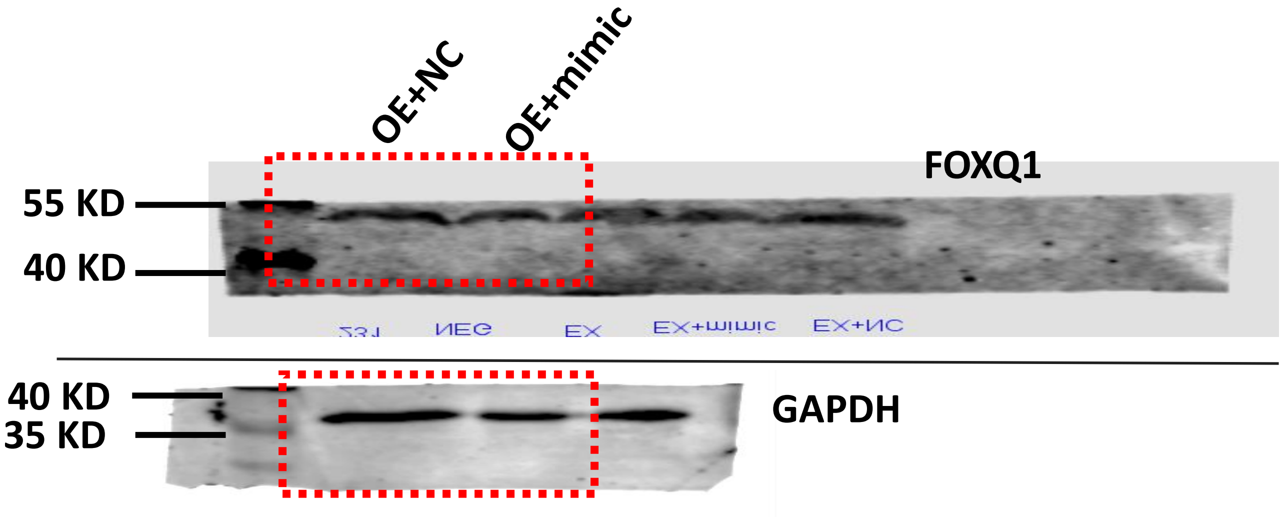


Figure 5h Hs578T

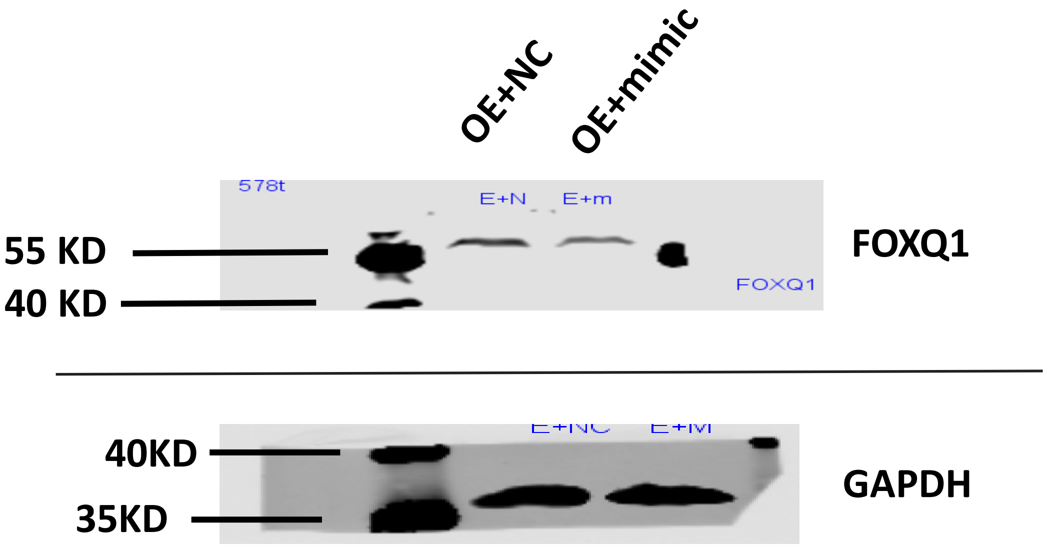

Supplement: Supplementary file 1 — Supplementary Material 1 [file 41598_2025_34859_MOESM1_ESM.pdf]
