# Supplementary material for: miR-96-5p antagonizes FOXQ1-driven WNT/β-catenin signaling to inhibit triple-negative breast cancer
Source: Sci Rep. 2026 Jan 4;16:4624. doi: 10.1038/s41598-025-34859-7 (PMC12868773; doi:10.1038/s41598-025-34859-7)
Supplement: Supplementary file 2 — Supplementary Material 2 [file 41598_2025_34859_MOESM2_ESM.pdf]

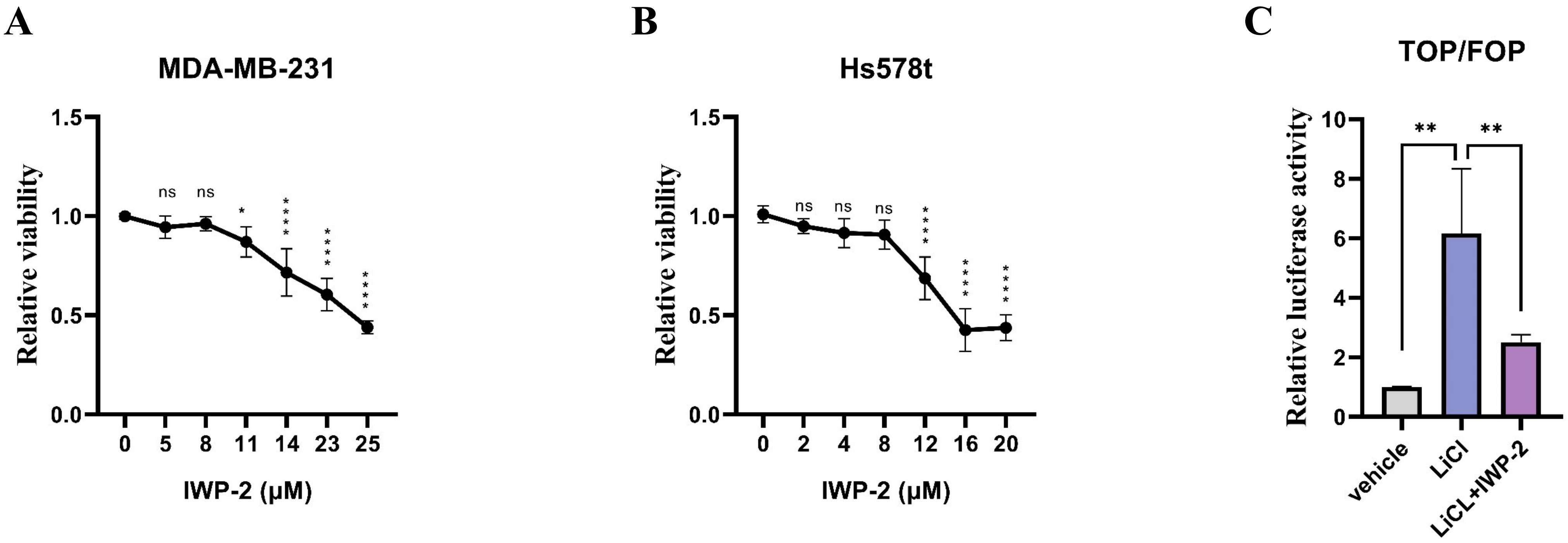

Figure S1 The concentration and on-target validation of IWP-2. A The relative viability of MDA-MB-231 cells was assessed following treatment with various concentrations of IWP-2. Cells in the logarithmic growth phase were seeded into 96-well plates at a density of 5,000 cells per well. Approximately 20 hours later, once cells had adhered and resumed proliferation, they were exposed to different concentrations of IWP-2 for an additional 24 hours, with six replicate wells per concentration group. Cell viability was then evaluated using the CCK-8 assay. **B** Relative viability of Hs578T cells after treatment with varying concentrations of IWP-2 was evaluated using the CCK-8 assay. Data were obtained from six replicate wells. **C** The relative luciferase activity of the TOP/FOP-Flash reporter system. HEK293T cells were transfected with either the TOP-Flash or FOP-Flash plasmid, together with a Renilla luciferase reporter plasmid for normalization. Following a 24-hour treatment with vehicle control, lithium chloride (LiCl), or a combination of LiCl and IWP-2, firefly luciferase activity was measured and normalized to Renilla luciferase activity to correct for variations in transfection efficiency and cell viability. The FOP-Flash construct was used as a negative control. Statistical results were derived from at least three independent experiments and are presented as mean ± standard deviation (SD). *, ***, ****, and ns denote *p* values < 0.05, 0.001, 0.0001, and non-significant, respectively, as determined by one-way ANOVA followed by Dunnett's multiple comparisons test for three or more groups or the student's t-test for two groups.
